# Natural Product Screening Reveals Naphthoquinone Complex I Bypass Factors

**DOI:** 10.1371/journal.pone.0162686

**Published:** 2016-09-13

**Authors:** Scott B. Vafai, Emily Mevers, Kathleen W. Higgins, Yevgenia Fomina, Jianming Zhang, Anna Mandinova, David Newman, Stanley Y. Shaw, Jon Clardy, Vamsi K. Mootha

**Affiliations:** 1 Howard Hughes Medical Institute and Department of Molecular Biology, Massachusetts General Hospital, Boston, MA, United States of America; 2 Broad Institute of MIT and Harvard, Cambridge, MA, United States of America; 3 Department of Biological Chemistry and Molecular Pharmacology, Harvard Medical School, Boston, MA, United States of America; 4 Department of Dermatology, Massachusetts General Hospital, Boston, MA, United States of America; 5 Natural Products Branch, National Cancer Institute, Frederick, MD, United States of America; 6 Center for Systems Biology, Massachusetts General Hospital, Boston, MA, United States of America; 7 Department of Systems Biology, Harvard Medical School, Boston, MA, United States of America; Broad Institute, UNITED STATES

## Abstract

Deficiency of mitochondrial complex I is encountered in both rare and common diseases, but we have limited therapeutic options to treat this lesion to the oxidative phosphorylation system (OXPHOS). Idebenone and menadione are redox-active molecules capable of rescuing OXPHOS activity by engaging complex I-independent pathways of entry, often referred to as “complex I bypass.” In the present study, we created a cellular model of complex I deficiency by using CRISPR genome editing to knock out *Ndufa9* in mouse myoblasts, and utilized this cell line to develop a high-throughput screening platform for novel complex I bypass factors. We screened a library of ~40,000 natural product extracts and performed bioassay-guided fractionation on a subset of the top scoring hits. We isolated four plant-derived 1,4-naphthoquinone complex I bypass factors with structural similarity to menadione: chimaphilin and 3-chloro-chimaphilin from *Chimaphila umbellata* and dehydro-α-lapachone and dehydroiso-α-lapachone from *Stereospermum euphoroides*. We also tested a small number of structurally related naphthoquinones from commercial sources and identified two additional compounds with complex I bypass activity: 2-methoxy-1,4-naphthoquinone and 2-methoxy-3-methyl-1,4,-naphthoquinone. The six novel complex I bypass factors reported here expand this class of molecules and will be useful as tool compounds for investigating complex I disease biology.

## Introduction

Complex I of the mitochondrial OXPHOS system is a macromolecular machine that simultaneously carries out two key activities: the transfer of electrons from mitochondrial matrix NADH to coenzyme Q (CoQ) and the pumping of protons across the inner mitochondrial membrane [1]. Breakdown of this complex results in broad biochemical defects, including NAD+/NADH ratio imbalance and impaired maintenance of the mitochondrial membrane potential. Mutations in structural subunits or assembly factors can severely impair complex I activity and are a common cause of mitochondrial disorders [2, 3]. Reduced complex I activity has also been implicated as a contributor to Parkinson’s disease, with data from both human genetics and model organism studies providing support [4].

One potential strategy for treating complex I deficiency is to rescue OXPHOS activity by engaging complex I-independent pathways of entry, often referred to as “complex I bypass.” Idebenone and menadione (vitamin K_3_) are two redox-active quinone compounds that are known to possess this activity [5–7], though most mechanistic studies have focused on idebenone. No mechanism has been identified whereby idebenone can accept electrons from the matrix NADH pool utilized by complex I. However, mitochondrial complex II, mitochondrial glycerol-3-phosphate dehydrogenase, and the cytosolic NAD(P)H quinone oxidoreductase 1 (NQO1) can convert idebenone to its reduced form, idebenol, which is subsequently oxidized by complex III thereby fueling the OXPHOS system [6, 8, 9]. Which one of these pathways of complex I bypass is dominant *in vivo* and whether additional routes exist remain open questions.

Idebenone, was recently approved in Europe for Leber’s Hereditary Optic Neuropathy (LHON), a genetic disorder most commonly due to mutations in mitochondrial DNA-encoded complex I structural subunits [10]. The evidence for idebenone’s efficacy is limited, however, and it was approved under “exceptional circumstances” with the requirement for continued evaluation of its clinical benefit [11]. Furthermore, there is uncertainty around the drug’s precise mechanism of action, which is proposed to be a combination of complex I bypass and antioxidant effects [12]. Expanding the class of complex I bypass factors may help to improve our understanding of idebenone’s mechanism of action and help to guide the development of more effective complex I therapeutics.

In the current study, we developed a chemical screening platform and used it to identify novel complex I bypass factors. We used CRISPR genome editing to generate a complex I deficient mouse myoblast cell line lacking the structural subunit *Ndufa9*, and used this cell line as the basis for a high-throughput complex I bypass assay. We then applied this screening assay to a collection of ~40,000 natural product extracts. Natural product extracts have historically been a rich source of therapeutic compounds [13]. In comparison to traditional libraries of “drug-like” compounds, we hypothesized that natural product extracts might contain a more diverse array of redox-active compounds that can serve as complex I bypass factors. Through our screening efforts and subsequent bioassay-guided fractionation we isolated four plant-derived 1,4-naphthoquinone compounds with complex I bypass activity. These compounds are structurally similar to menadione, and testing of related compounds obtained from commercial suppliers enabled us to identify two additional complex I bypass factors. These results validate our screening platform and introduce a new set of six complex I bypass factors.

## Materials and Methods

### Cell Culture

C2C12 myoblasts were obtained from ATCC (CRL-1772) and were grown in high glucose (4.5 g/L) Dulbecco’s Modified Eagle Medium (DMEM, Gibco) supplemented with 2 mM GlutaMAX, 10% (vol/vol) fetal bovine serum (FBS), 100 U/mL penicillin, and 100 μg/mL streptomycin. *Ndufa9* knockout cells were grown in the same media supplemented with 50 μg/mL uridine. All cells were grown in a humidified incubator with 5% CO2 at 37°C. Cells were maintained at low density and all assays were performed between four and fifteen passages after thawing.

### Generation of *Ndufa9* Knockout Cell Line

The second exon of *Ndufa9* was targeted with the CRISPR/Cas9 system using the guide sequence GGTTAACAACGTATCGACCC, which was selected from a UCSC genome browser track generated by the Zhang Lab. This guide sequence was ligated into the vector pX330 [14] and transfected into C2C12 cells using Amaxa Cell Line Nucleofector Kit V (Lonza). The transfected cells were allowed to recover for three days, and then were seeded in 96-well plates at a concentration of 0.5 cells per well. The resulting single cell clones were evaluated by Western blotting against NDUFA9 (Abcam, ab14713). For the Western blot displayed here, anti-β-tubulin (Cell Signaling, 2128) was used as a loading control. The *Ndufa9* knockout clone used in this paper was further validated by next-generation sequencing. The genomic region targeted by CRISPR was PCR-amplified and tagged with partial Illumina adapter sequences using the following primers: forward (TCGTCGGCAGCGTCAGATGTGTATAAGAGACAGNNNNNNTCCTAGTCCAAGGCATGAAGC) and reverse (GTCTCGTGGGCTCGGAGATGTGTATAAGAGACAGNNNNNNGCCTCTGGTGTTTCCCTGAA). The PCR product was purified with AMPure XP beads (Agencourt), and further amplified using the Advantage 2 PCR kit. In this second round of PCR, primers from the Nextera Index Kit (Illumina) were used to add complete Illumina sequencing adapters and sample barcodes. The final PCR product was purified with the QIAquick PCR Purification Kit (Qiagen) and sequenced on a MiSeq (Illumina).

### Complex I and IV Dipstick Assays

The enzymatic activities of OXPHOS complex I and IV were determined using commercial dipstick assays from Abcam (ab109720 for complex I and ab109878 for complex IV), following the manufacturer’s protocols. Assays for complex I and IV activity were performed with 50 μg and 100 μg of total protein per dipstick, respectively.

### Seahorse Bioenergetic Analysis

Oxygen consumption rates were measured with an XF96 Analyzer and an XFe96 Analyzer (Seahorse Biosciences) using a modified version of the manufacturer’s protocol. On the day prior to the assay, cells were seeded on plates pre-treated with Cell Tak (Corning) at a density of 12,000 cells/well in 80 μL of our standard cell culture media described above and incubated overnight. On the day of the experiment, the cell plate was washed with Seahorse assay media: low-glucose (1 g/L), bicarbonate-free DMEM (U.S. Biological, D9800) supplemented with 2 mM GlutaMAX, penicillin/streptomycin, 50 μg/mL uridine and adjusted to pH 7.4. For rotenone pre-treatment experiments, the assay media also contained 500 nM rotenone and 25 mM HEPES buffer. Inhibitors and extracts were dissolved in DMSO and diluted in media prior to loading into the chemical cartridge. The cell plate and the loaded chemical cartridge were both allowed to equilibrate in an ambient-CO2, 37°C incubator for approximately thirty minutes before the start of the experiment. We programmed the machine to sequentially inject 5–8 μM oligomycin, 2–4 μM CCCP, and 1 μM antimycin (all from Sigma and dissolved in DMSO), separated by periods of mixing and measurement. When testing extracts or pure compounds for bypass activity, they were injected early in the experiment, before the three inhibitors. For all Seahorse experiments shown in this paper, at least six wells were used for each condition and the average of these technical replicates is shown. Once the program completed, two rows of each cell type were trypsinized, separately pooled, and counted in triplicate. The average of the two rows was used to normalize raw Seahorse data. While analyzing Seahorse data, we omitted as outliers any wells with raw oxygen levels less than 100 mmHg or greater than 200 mmHg for the first tick of the first three cycles during the baseline measurement phase. No more than six experimental wells needed to be excluded in any of the experiments included in this paper.

### Luminescence-Based Complex I Bypass Assay

The day prior to the assay, C2C12 *Ndufa9* knockout cells were seeded in 384-well plates in DMEM containing 3 mM glucose supplemented with penicillin/streptomycin, 2 mM GlutaMAX, 10% FBS, and 50 μg/mL uridine at a density of 3,750 cells/well in 50 μL of media. Approximately one day later, the plate was washed three times with PBS and the media was replaced with DMEM containing no glucose or FBS. Test compounds were added using a HP D300 Digital Dispenser. For experiments involving antimycin, 125 nM antimycin was added after the test compound. Cells were incubated at 37°C for 45–90 minutes and then ATP content was measured using the Cell Titer Glo assay (Promega). The Cell Titer Glo reagent was prepared according to the manufacturer’s protocol and then diluted three-fold in PBS. Fifteen μL of diluted reagent were added to each well, and the plate was allowed to incubate for 10 minutes at room temperature. Following incubation, luminescence was measured using a Tecan plate reader. The luminescence of each well was normalized by expressing it as a fold-change relative to the average luminescence of the appropriate negative control wells on the same plate.

### Natural Product Screen

The complex I bypass assay described above was used to screen a library of 39,510 natural product extracts that was obtained from the Natural Products Branch of the National Cancer Institute (NCI). This library contains nearly equal representation of plant extracts, extracts from marine organisms, and extracts derived from microorganisms. Extracts were transferred from 384-well compound plates to cell plates using disposable Pin Sterile Replicators (Phenix Research Products). Each compound plate contained DMSO-solubilized extracts as well as negative control wells that were either empty or contained DMSO. The luminescence of each extract well was normalized to the average of all negative control wells. On each of the 15 screening days, an additional control plate was tested that contained several doses of idebenone and was normalized to the average luminescence of DMSO control wells.

### Bioassay-Guided Isolation of Active Metabolites from the NCI Extracts

Among the extracts that scored highly in our primary screen of the natural product library, 13 also passed additional screening as described in the results section. Seven of these were chosen for further fractionation by reverse-phase solid phase extraction (SPE). We applied approximately 100 mg of extract to a stepwise gradient solvent system of decreasing polarity, transitioning from 75% H_2_O/acetonitrile (ACN) to 100% dichloromethane (DCM). This yielded five fractions, which we refer to as “SPE A-E.” All SPE fractions were subsequently tested in the Seahorse assay and the metabolites in the active fractions were profiled by liquid chromatography mass spectrometry (LCMS) analysis. For five extracts, the SPE C fraction (75% ACN/H_2_O) displayed inhibitor sensitive increases in oxygen consumption in the Seahorse assay. All of these extracts were produced by plants, four belonging to the *Pyrolaceae* family, including three from *Chimaphila umbellata*. The fifth extract was derived from *Stereospermum euphoroides*.

Active components from one of the *C*. *umbellata* SPE C fractions were purified using a Phenomenex 4 μm Hydro semi-preparative column with the following gradient: hold 40% ACN + 0.1% formic acid (FA)/H_2_O + 0.1% FA from 0–5 min, gradient to 100% ACN + 0.1% FA over 35 min. This yielded pure chimaphilin, 3-chloro-chimaphilin, and 3-hydroxy-chimaphilin (S1 Appendix). An alternative source of these metabolites was obtained by purifying a commercially available herbal extract of the leaves of *C*. *umbellata* (Pipsissewa extract from Herb Pharm).

The active metabolites in the *S*. *euphoroides* extract were purified using a Phenomenex Kinetex Biphenyl semi-preparative column, with the following gradient: hold 30% ACN/H_2_O from 0–5 min, then gradient to 65% ACN/H_2_O over 30 min, yielding pure α-lapachone, lapachol, dehydro-α-lapachone, and a racemic mixture of dehydroiso-α-lapachone (S2 Appendix). Following standard LCMS evaluation, the structures of dehydro-α-lapachone and lapachol were further verified by comparison of the natural product with standards purchased from Sigma. All subsequent assays were conducted with the commercially supplied material. Three structurally related naphthoquinone compounds (menadione, 2-methoxy-1,4-naphthoquinone and 2-methoxy-3-methyl-1,4,-naphthoquinone) were obtained from Sigma for additional testing.

**Chimaphilin (1):** pale yellow solid; UV (MeOH) *λ*_max_ (log ε) 206 (3.79), 250 (3.97), 255 (3.98), 338 (3.14) nm; ^1^H NMR (600 MHz, CDCl_3_) 7.82 (1H, d; 7.8), 7.77 (1H, s), 7.42 (1H, d; 7.8), 6.69 (1H, s), 2.38 (3H, s), 2.06 (3H, s); ^13^C NMR (150 MHz, CDCl_3_) 185.9, 184.5, 148.2, 144.6, 135.7, 134.3, 132.0, 131.1, 126.8, 126.2, 21.8, 16.4; HRESIMS [M+H]^+^ *m/z* 187.0752 (calcd for C_12_H_11_O_2_ 187.0759, Δ 3.7 ppm).

**3-Hydroxy-chimaphilin (2):** yellow amorphous solid; UV (MeOH) *λ*_max_ (log ε) 205 (3.64), 253 (3.62), 257 (3.66), 287 (3.53), 330 (2.80) nm; ^1^H NMR (600 MHz, CDCl_3_) 7.97 (1H, d; 7.8), 7.91 (1H, s), 7.46 (1H, d; 7.8), 2.49 (3H, s), 2.09 (3H, s); ^13^C NMR (150 MHz, CDCl_3_) 185.5, 181.5, 153.5, 145.2, 133.4, 132.8, 127.2, 127.0, 120.0, 22.1, 8.6; HRESIMS [M+H]^+^ *m/z* 203.0699 (calcd for C_12_H_11_O_3_ 203.0708, Δ 4.4 ppm).

**3-Chloro-chimaphilin (3):** pale yellow amorphous solid; UV (MeOH) *λ*_max_ (log ε) 205 (3.50), 251 (3.36), 256 (3.37), 272 (3.27), 278 (3.29), 340 (2.59) nm; ^1^H NMR (600 MHz, CDCl_3_) 8.05 (1H, d; 8.0), 7.91 (1H, s), 7.53 (1H, d; 8.0), 2.50 (3H, s), 2.34 (3H, s); ^13^C NMR (125 MHz, CDCl_3_) 182.9, 177.4, 145.4, 144.6, 143.4, 134.6, 131.6, 129.1, 127.4 x2, 21.9, 14.5; HRESIMS [M+H]^+^ *m/z* 221.0364 (calcd for C_12_H_10_ClO_2_ 221.0369, Δ 2.2 ppm).

**Lapachol (4):** red amorphous solid; UV (MeOH) *λ*_max_ (log ε) 209 (4.04), 252 (4.11), 280 (3.94), 331.0 (3.29) nm; ^1^H NMR (600 MHz, CDCl_3_) 8.11 (1H, d; 7.6), 8.06 (1H, d; 7.7), 7.74 (1H, t; 7.4), 7.67 (1H, t; 7.7), 5.20 (1H, t; 7.3), 3.30 (2H, d; 7.3), 1.79 (3H, s), 1.68 (3H, s); ^13^C NMR (150 MHz, CDCl_3_) 184.5, 181.7, 152.7, 134.8, 133.8, 132.9, 132.8, 129.4, 126.7, 126.0, 123.5, 119.6, 25.7, 22.6, 17.9; HRESIMS [M+H]^+^ *m/z* 243.1018 (calcd for C_15_H_15_O_3_ 243.1021, Δ 1.2 ppm).

**Dehydro-α-lapachone (5):** yellow amorphous solid; UV (MeOH) *λ*_max_ (log ε) 206 (3.97), 259 (3.76), 280 (3.73), 328 (3.10) nm; ^1^H NMR (600 MHz, CDCl_3_) 8.09 (2H, d; 7.4), 7.71 (1H, t; 7.2), 7.68 (1H, t; 7.3), 6.66 (1H, d; 10.1), 5.73 (1H, d; 10.1), 1.56 (6H, s); ^13^C NMR (150 MHz, CDCl_3_) 184.3, 179.9, 152.4, 134.0, 133.2, 131.6, 131.5, 130.9, 126.2x2, 117.9, 115.5, 80.4, 28.4x2; HRESIMS [M+H]^+^ *m/z* 241.0860 (calcd for C_15_H_13_O_3_ 241.0865, Δ 2.9 ppm).

**α-Lapachone (6):** yellow amorphous solid; UV (MeOH) *λ*_max_ (log ε) 206 (3.70), 251 (3.89), 282 (3.66), 332 (3.00) nm; ^1^H NMR (600 MHz, CDCl_3_) 8.08 (2H, t; 8.3), 7.70 (1H, t; 7.2), 7.6 6 (1H, t; 7.8), 2.62 (2H, t; 6.6), 1.82 (2H, t; 6.6), 1.44 (6H, s); ^13^C NMR (125 MHz, CDCl_3_) 184.4, 180.0, 154.6, 133.8, 132.9, 132.1, 131.2, 126.3, 126.0, 120.2, 78.2, 31.5, 26.5 x 2, 16.8; HRESIMS [M+H]^+^ *m/z* 243.1017 (calcd for C_15_H_15_O_3_ 243.1021, Δ 1.6 ppm).

**Dehydroiso-α-lapachone (7):** pale yellow amorphous solid; UV (MeOH) *λ*_max_ (log ε) 205 (3.84), 248 (3.97), 253 (4.01), 288 (3.73), 333 (3.12) nm; ^1^H NMR (600 MHz, CDCl_3_) 8.09 (2H, t; 8.4), 7.73 (1H, t; 7.4), 7.68 (1H, t; 7.6), 5.42 (1H, dd; 10.4, 8.9), 5.13 (1H, s), 5.00 (1H, s), 3.36 (1H, dd; 17.1, 10.9), 3.04 (1H, dd; 17.1, 8.8), 1.81 (3H, s); ^13^C NMR (125 MHz, CDCl_3_) 182.2, 177.7, 160.0, 141.6, 134.2, 133.0, 131.6 x 2, 126.3, 126.0, 124.0, 113.9, 88.5, 32.0, 16.9; HRESIMS [M+H]^+^ *m/z* 241.0863 (calcd for C_15_H_13_O_3_ 241.0865, Δ 0.4 ppm).

## Results

### Platform for Complex I Bypass Screening

**Fig 1:**
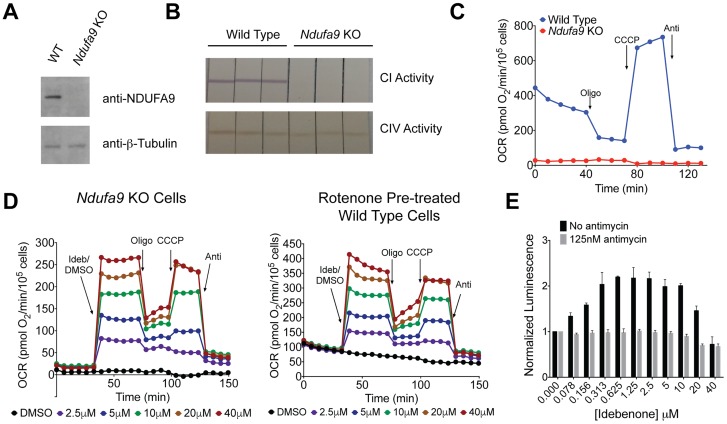
Validation of Complex I Deficient Cell Line and Bypass Screening Assay. (A) Western blot of wild-type and *Ndufa9* knockout C2C12 cells. (B) Complex I and complex IV dipstick assays for wild-type and *Ndufa9* knockout C2C12 cells, with technical triplicates shown for each. (C) Seahorse comparing wild-type and *Ndufa9* knockout C2C12 cells. (D) Seahorse of *Ndufa9* knockout cells (left) and rotenone pre-treated wild-type C2C12s (right), each treated with a dose-response of idebenone. Doses are indicated in the legend below each plot. (E) Luminescence-based complex I bypass assay utilizing *Ndufa9* knockout cells. Cells treated with a dose-response of idebenone in the presence or absence of 125 nM antimycin. For (A-D), each experiment was performed three times, and a single representative experiment is shown. For (E) the data shown represent the average +/- SEM of data from three independent experiments.

In order to model complex I deficiency, we utilized the CRISPR genome editing system to generate a clonal C2C12 mouse myoblast cell line harboring loss-of-function mutations in the structural subunit *Ndufa9*. We chose to target this specific gene because mutations in it have been reported to cause severe mitochondrial disease in humans [15]. In addition, knockout of this gene using TALEN technology in HEK293 cells has been shown to cause complex I deficiency, while leaving complexes III and IV intact [16]. In our *Ndufa9* knockout cell line, we did not detect any remaining NDUFA9 protein by Western blot (Fig 1A). Next-generation sequencing of the targeted locus indicated the presence of four unique deletion mutations predicted to disrupt the function of the gene (S1 Fig). No wild-type sequences were detected. The presence of four unique mutations suggests that the cell line either has four mutant copies of *Ndufa9*, or that there are two cell lines present that are each diploid at this locus. In either case, the population of cells is predicted to have no functional copies of this gene and provides a useful model of complex I deficiency for therapeutics discovery.

We used enzymatic assays to evaluate two OXPHOS complexes in our *Ndufa9* knockout cell line and found that complex I activity is undetectable, while complex IV activity is present (Fig 1B), consistent with the published HEK293 cell knockout of this gene [16]. The knockout cell line also demonstrated near complete absence of oxygen consumption, even following treatment with the uncoupler CCCP (Fig 1C). One prior study demonstrated that idebenonol is capable of rescuing OXPHOS activity in a cellular genetic model of complex I deficiency [5]. We evaluated the effects of idebenone using two different models of complex I deficiency: wild-type cells pre-treated with the complex I inhibitor rotenone and our *Ndufa9* knockout cell line. We found that treatment with idebenone stimulates oxygen consumption in both models of complex I deficiency (Fig 1D). Furthermore, idebenone’s effect is sensitive to classical OXPHOS inhibitors, supporting a mitochondrial mechanism of action: the rate of oxygen consumption decreases with the addition of oligomycin (complex V inhibition), increases with CCCP, and finally falls with antimycin treatment (complex III inhibition). This supports the hypothesis that in the absence of complex I, idebenone allows “bypass” by shuttling electrons from alternative donors to complex III. In addition, these data indicate that the OXPHOS system distal to complex I remains intact in our knockout cell line. Finally, the close agreement between idebenone’s effects in our chemical and genetic models further validates our knockout cell line as a model of complex I deficiency.

We next sought to develop a screening platform to discover additional compounds with complex I bypass activity in an unbiased manner. Haefeli *et al*. previously reported a high-throughput complex I bypass assay in which cells are placed in glucose-free media, forcing them to utilize OXPHOS for ATP production [6]. Rotenone is added to block complex I, and the ability of a test compound to bypass this inhibition is read out by an endpoint measurement of ATP after one hour of treatment. Idebenone demonstrates bypass activity in this assay, as do several related quinones [17]. We utilized our genetic model of complex I deficiency in a modified version of the Haefeli *et al*. assay, and confirmed that idebenone increases ATP levels (Fig 1E). This increase in ATP is sensitive to antimycin, consistent with idebenone’s proposed mechanism of complex I bypass.

### Natural Product Screening Reveals Novel Complex I Bypass Factors

**Fig 2:**
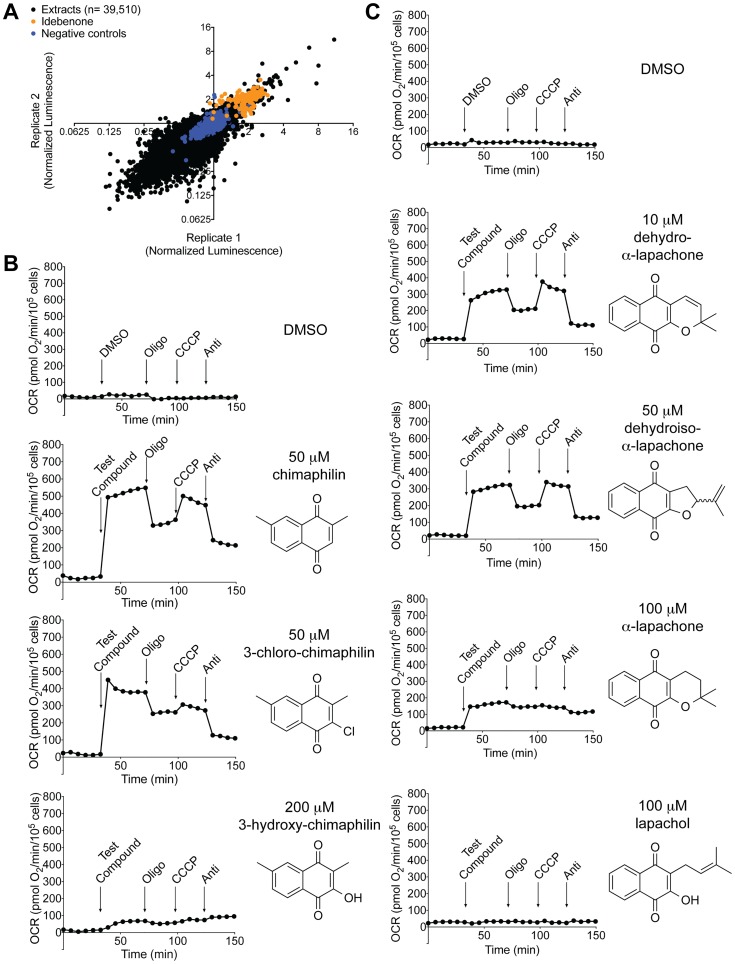
Natural Product Screen and Follow-up. (A) Scatter plot of the normalized luminescence scores for the two original replicates of the natural product screening data from S1 Table (see S2 Table for data from the plates retested with and without antimycin). The data are plotted on a Log2 scale. The extract plates included DMSO wells and empty wells, which are plotted as the “negative controls.” The positive control plates contained wells with 2.4 mM idebenone, which was diluted upon transfer to the cell plate. These points are labeled “idebenone.” (B) Seahorse data for the three compounds isolated from *Chimaphila umbellata*. (C) Seahorse data for the four compounds isolated from *Sterospermum euphoroides*. For (B) and (C), each experiment was performed three times and data from a single representative experiment is shown.

In order to discover novel complex I bypass factors, we applied the high-throughput complex I bypass assay described above to a library of approximately 40,000 diverse extracts from bacteria, plants, fungi, and marine organisms created by the Natural Products branch of the NCI. The results of the screen are detailed in S1 and S2 Tables and displayed in Fig 2A.

The natural product extract screen was carried out over the course of 15 days. Each plate was tested in duplicate on the same day, and we generally observed good agreement between replicates. However, there was a high degree of variability in the magnitude of the idebenone positive control signal between days, which made it difficult to directly compare results from different days. In order to prioritize extracts for follow-up, we used a three-stage nomination process: analysis of individual screening days, further testing of plates with many high-scoring extracts, and consideration of full-screen data. For each screening day, we plotted extract scores alongside the positive and negative controls from the same day, and nominated extracts with either a high average score or a very high single score relative to the positive control signal distribution. On three days, an unusually large number of extracts scored within or above the positive control range. We retested the plates containing these high scoring extracts on another screening day. On the retest, we also included a condition with antimycin treatment (data provided in S2 Table). Extracts that consistently scored highly on both days relative to the positive control idebenone distribution and/or demonstrated any sensitivity to antimycin were added to the list of hits. Finally, we compiled a rank-ordered list of all ~40,000 extracts (S1 Table). Most of the top-ranked hits had already been nominated, but we added several additional extracts that fell within or above the aggregate group of positive control data points from all screening days. Finally, we eliminated one extract that had been previously evaluated by our laboratory and showed no capacity to increase oxygen consumption in Seahorse analysis. Based on these criteria, we ultimately nominated 159 extracts for follow-up, 132 of which were available from NCI for further analysis.

Among the 132 extracts received for follow-up, 121 were retested in our primary luminescence-based bypass assay in dose response. The remaining 11 extracts were evaluated in Seahorse, along with all extracts with a normalized score of at least 1.5 at any dose in the retest. In a typical Seahorse experiment, we utilize the response to OXPHOS inhibitors to determine if changes in oxygen consumption are due to complex I bypass. However, these crude extracts are presumed to contain a mixture of compounds that may affect oxygen consumption in multiple ways. For screening purposes, we considered an extract to be a hit if it increased oxygen consumption upon acute treatment, regardless of inhibitor sensitivity. Of the 132 extracts received from NCI, only 13 met these criteria. Of note, five of these extracts were derived from flowering plants in the same family, *Pyrolaceae*: three came from *Chimaphila umbellata*, one from *Pyrola asarifolia*, and one from *Pyrola secunda*.

We used the Seahorse assay to guide the iterative fractionation of one of the *C*. *umbellata* extracts. These efforts ultimately yielded a family of closely related 1,4-naphthoquinone compounds: chimaphilin, 3-hydroxy-chimaphilin, and 3-chloro-chimaphilin. Data for all three compounds are shown in Fig 2B. Chimaphilin and 3-chloro-chimaphilin both cause oligomycin and antimycin sensitive increases in oxygen consumption, indicating that they have complex I bypass activity. In contrast, 3-hydroxy-chimaphilin causes only a slight increase in oxygen consumption that is not sensitive to OXPHOS inhibitors, suggesting that it does not possess complex I bypass activity. We used LCMS to confirm the presence of chimaphilin and 3-hydroxy-chimaphilin in the four additional *Pyrolaceae* extracts, including extracts from *P*. *asarifolia* and *P*. *secunda*, and two more from *C*. *umbellata*. We suspect that 3-chloro-chimaphilin is also present in these samples, but is below the limits of detection. Of note, chimaphilin, 3-hydroxy-chimaphilin, and 3-chloro-chimaphilin are all structurally similar to menadione, a 1,4-naphthoquinone compound that is known to have complex I bypass activity [7, 17]. We verified the complex I bypass activity of menadione in the Seahorse assay, and also observed comparable effects with two closely related naphthoquinone compounds (2-methoxy-1,4-naphthoquinone and 2-methoxy-3-methyl-1,4,-naphthoquinone), neither of which has been previously shown to have bypass activity (S2 Fig).

Bioassay-guided fractionation of the Seahorse-active extract from *Stereospermum euphoroides* yielded an additional set of naphthoquinone compounds: lapachol, α-lapachone, dehydro-α-lapachone, and dehydroiso-α-lapachone (Fig 2C). Among these compounds, dehydro-α-lapachone and dehydroiso-α-lapachone show Seahorse profiles consistent with complex I bypass, with responses to all three OXPHOS inhibitors. Lapachol does not affect oxygen consumption, suggesting that it does not interact with the OXPHOS system. Although α-lapachone increases oxygen consumption, this effect is only modestly sensitive to oligomycin and antimycin and shows no response to CCCP. As such, we do not believe that α-lapachone possesses significant complex I bypass activity. The lack of activity of α-lapachone, lapachol, and 3-hydroxy-chimaphilin will help to guide any future medicinal chemistry efforts to improve compound efficacy around the 1,4-naphthoquinone scaffold.

Besides the extract from *S*. *euphoroides* and the five extracts from the *Pyrolaceae* family, we identified seven additional extracts that increased oxygen consumption in our *Ndufa9* knockout cells. Four were studied further, but showed a lack of promise for yielding complex I bypass factors (summarized in S1 Table**)**. The remaining extracts from *Gliocladium roseum* and two actinomycete species increased oxygen consumption in Seahorse, but were not studied in depth. These extracts could contain active compounds and merit follow-up in future studies.

## Discussion

In the present study, we utilized CRISPR to engineer a cellular disease model of complex I deficiency, which served as the foundation for a complex I bypass factor screening platform. We applied this platform to a large library of natural product extracts and discovered four 1,4-naphthoquinone complex I bypass factors from two plant species, *C*. *umbellata* and *S*. *euphoroides*. In addition, we tested several commercially available compounds with structural similarity to our screening hits and demonstrated that they can also function as complex I bypass factors. Given their structural similarity to menadione, we hypothesize that these compounds accept electrons from various quinone-reducing enzymes and deliver them to complex III. Future mechanistic studies should focus on defining the full spectrum of enzymes capable of mediating complex I bypass by quinone compounds and determining their individual importance in both normal and disease physiology. We anticipate that the expanded toolkit of complex I bypass factors presented here will be enabling for these experiments.

The flowering plant *C*. *umbellata* has been used widely as an herbal medicine. For example, it has been used by Canadian First Nations peoples for a variety of purposes, including as a diuretic and as a treatment for the common cold [18]. *C*. *umbellata* extract is also included in Eviprostat, an herbal treatment for benign prostatic hypertrophy [19]. We have found that an extract from this plant contains at least two 1,4-naphthoquinone compounds with complex I bypass activity: chimaphilin and 3-chloro-chimaphilin. Although chimaphilin is a well-documented component of *C*. *umbellata* [20], it has never been studied in relation to mitochondrial dysfunction. One intriguing question raised by our study is whether any of the clinical effects attributed to *C*. *umbellata* extract might be at all related to its impact on mitochondrial function.

*S*. *euphoroides* is not as well studied as *C*. *umbellata* and lacks a significant history of use in herbal medicine. We found that an extract from this plant contains at least two 1,4-naphthoquinone complex I bypass factors: dehydro-α-lapachone and dehydroiso-α-lapachone. To our knowledge, no biological activities have previously been attributed to dehydroiso-α-lapachone. However, dehydro-α-lapachone was found in one study to have “antivascular” activity and reduce tumor growth in a mouse model [21]. The results of our study suggest that the contribution of dehydro-α-lapachone’s complex I bypass activity to its anti-tumor effects should be explored further.

One potential liability of 1,4-naphthoquinones is the fact that they can promote the generation of reactive oxygen species (ROS) with resulting cytotoxicity, as has been demonstrated for menadione, chimaphilin, and 2-methoxy-1,4-naphthoquinone [22–24]. However, menadiol diphosphate, a precursor of menadione, has been used in combination with ascorbate for “bypass” treatment of a patient with complex III deficiency, without any toxic effects reported [25, 26]. Among the novel complex I bypass factors described here, dehydro-α-lapachone has been administered to mice for up to 16 days without causing any overt signs of toxicity [21]. Furthermore, menaquinone (vitamin K_2_), which differs from menadione by the presence of a polyisoprenoid side chain, has been shown to provide phenotypic improvement and bioenergetic rescue of a *Drosophila melanogaster* model of Parkinson’s disease known to have complex I deficiency [27, 28]. Though toxicity resulting from ROS production may limit the dose that can be used, we propose that the novel complex I bypass factors identified in this study should also be evaluated in complex I disease models.

In summary, we have used a CRISPR-engineered complex I deficient cell line to design a screening platform, which enabled us to identify four natural product 1,4-naphthoquinone complex I bypass factors. By searching for structurally similar compounds to our screening hits, we also uncovered two additional 1,4-naphthoquinone complex I bypass factors. Our screening approach could be readily applied to additional synthetic and natural product libraries in order further expand the number of available complex I bypass factors. Although their capacity to produce ROS may limit their development as therapeutics, we anticipate that the compounds described here will be useful as chemical tools to probe the role of complex I deficiency in disease models.

## Supporting Information

S1 Appendix This file contains analytical data on the chimaphilin family of metabolites, including ^1^H NMR, ^13^C NMR, and HRESIMS spectra for chimaphilin (1), 3-hydroxy-chimaphilin (2), and 3-chloro-chimaphilin (3). (PDF)

S2 Appendix This file contains analytical data on the lapachone family of metabolites, including ^1^H NMR, ^13^C NMR, and HRESIMS spectra for lapachol (4), dehydro-α-lapachone (5), α-lapachone (6), and dehydroiso-α-lapachone (7). Also included is the chiral HPLC chromatogram for dehydroiso-α-lapachone **(7)**.
(PDF)

S1 Fig Sequencing data for the *Ndufa9* knockout cell line. (TIF)

S2 Fig Seahorse experiment for menadione, 2-methoxy-1,4-naphthoquinone and 2-methoxy-3-methyl-1,4,-naphthoquinone. This experiment was performed three times and a single representative experiment is shown.
(TIF)

S1 Table Primary data for a screen of ~40,000 natural product extracts using our luminescence-based complex I bypass assay. For extracts that were retested, only the first trial is included. Please see S2 Table for full data for retested extracts.
(XLSX)

S2 Table This table contains the data for the extract plates that were retested in the presence or absence of 125 nM antimycin. The data from the original testing day (“Trial 1”) is included for reference alongside the data from retesting (“Trial 2”).
(XLSX)
