# Supplementary material for: Natural Product Screening Reveals Naphthoquinone Complex I Bypass Factors
Source: PLoS One. 2016 Sep 13;11(9):e0162686. doi: 10.1371/journal.pone.0162686 (PMC5021346; doi:10.1371/journal.pone.0162686)

**S1 Appendix**

**Analytical Data for the Chimaphilin Family of Metabolites**

**NMR Spectroscopic Data:**


**Figure S1** $^{1}$H NMR (600 MHz, $CDCl_3$) spectrum of chimaphilin (**1**)
**Figure S2** $^{13}$C NMR (150 MHz, $CDCl_3$) spectrum for chimaphilin (**1**)
**Figure S3** $^{1}$H NMR (600 MHz, $CDCl_3$) spectrum of 3-hydroxy-chimaphilin (**2**)
**Figure S4** $^{13}$C NMR (150 MHz, $CDCl_3$) spectrum for 3-hydroxy-chimaphilin (**2**)
**Figure S5** $^{1}$H NMR (600 MHz, $CDCl_3$) spectrum of 3-chloro-chimaphilin (**3**)
**Figure S6** $^{13}$C NMR (125 MHz, $CDCl_3$) spectrum for 3-chloro-chimaphilin (**3**)
**Figure S7** gHSQC ($^{1}$H 500 MHz, $CDCl_3$) spectrum for 3-chloro-chimaphilin (**3**)
**Figure S8** gCOSY ($^{1}$H 500 MHz, $CDCl_3$) spectrum for 3-chloro-chimaphilin (**3**)
**Figure S9** gHMBC ($^{1}$H 500 MHz, $CDCl_3$) spectrum for 3-chloro-chimaphilin (**3**)
**Table S1** $^{1}$H and $^{13}$C NMR data table for 3-chloro-chimaphilin (**3**) in $CDCl_3$
**Table S2** $^{1}$H and $^{13}$C NMR assignments for chimaphilin (**1**), 3-hydroxy-chimaphilin (**2**), and 3-chloro-chimaphilin (**3**) in $CDCl_3$


**HRESIMS Data:**


**Figure S10** HRESIMS spectrum for chimaphilin (**1**)
**Figure S11** HRESIMS spectrum for 3-hydroxy-chimaphilin (**2**)
**Figure S12** HRESIMS spectrum for 3-chloro-chimaphilin (**3**)

**S1:** $^{1}$H NMR (600 MHz, $CDCl_3$) spectrum of chimaphilin (**1**)

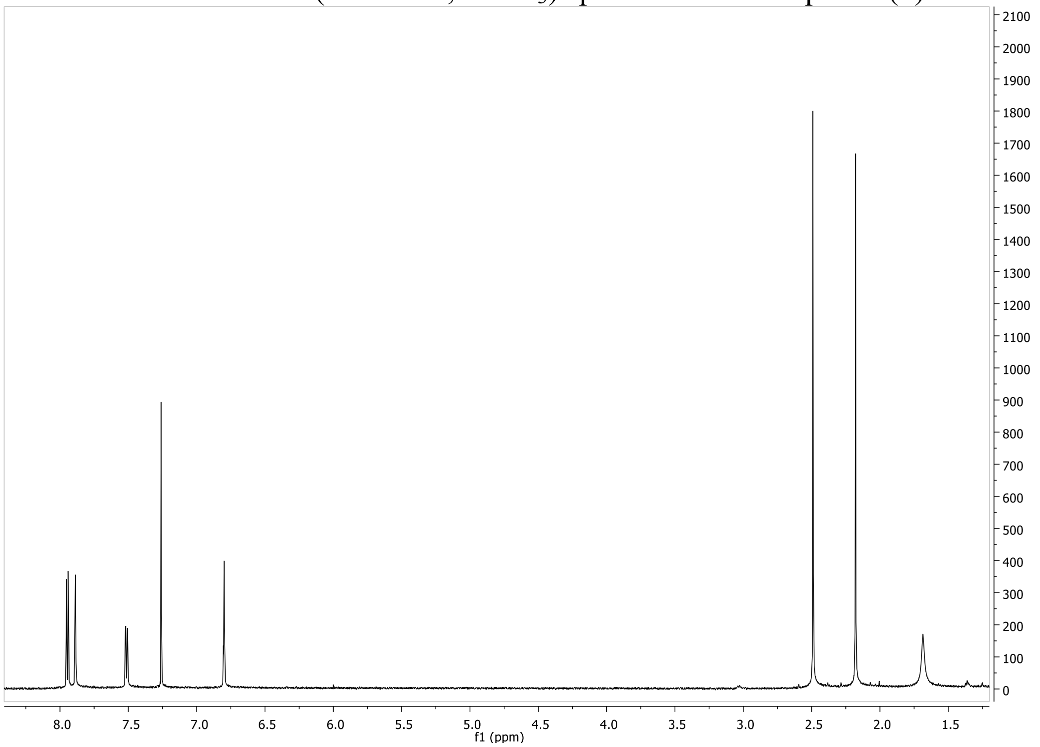


**S2:** $^{13}$C NMR (150 MHz, $CDCl_3$) spectrum for chimaphilin (**1**)

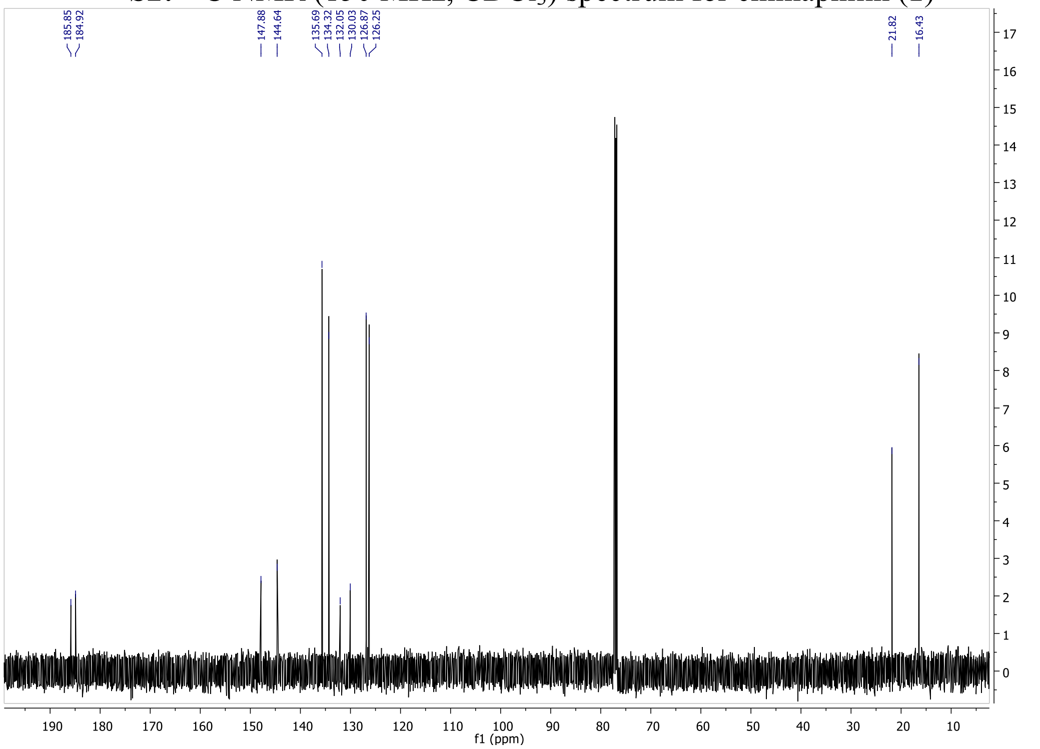

**S3:** $^{1}$H NMR (600 MHz, $CDCl_3$) spectrum of 3-hydroxy-chimaphilin (**2**)

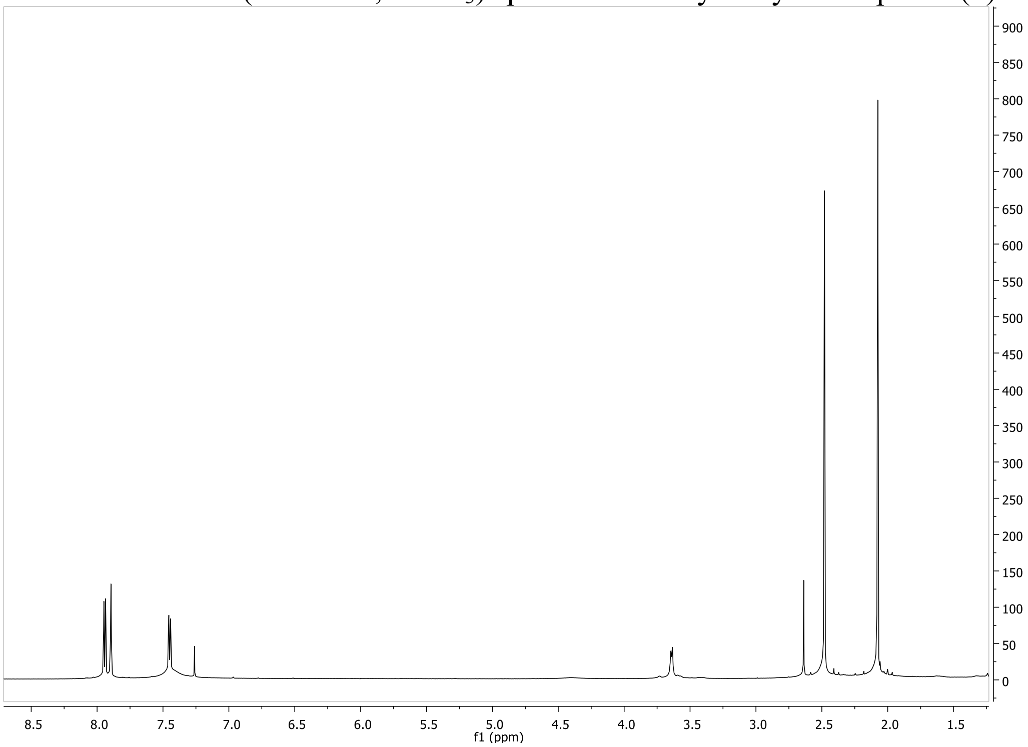


**S4:** $^{13}$C NMR (150 MHz, $CDCl_3$) spectrum for 3-hydroxy-chimaphilin (**2**)

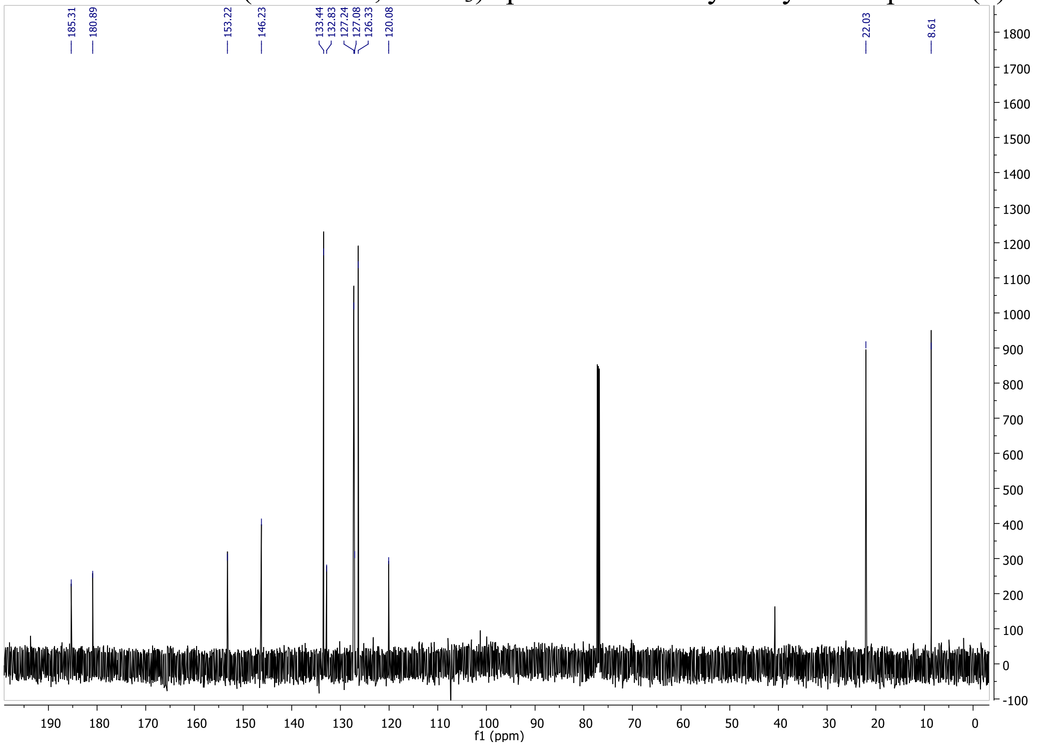

**S5:** $^{1}$H NMR (600 MHz, $CDCl_3$) spectrum of 3-chloro-chimaphilin (**3**)

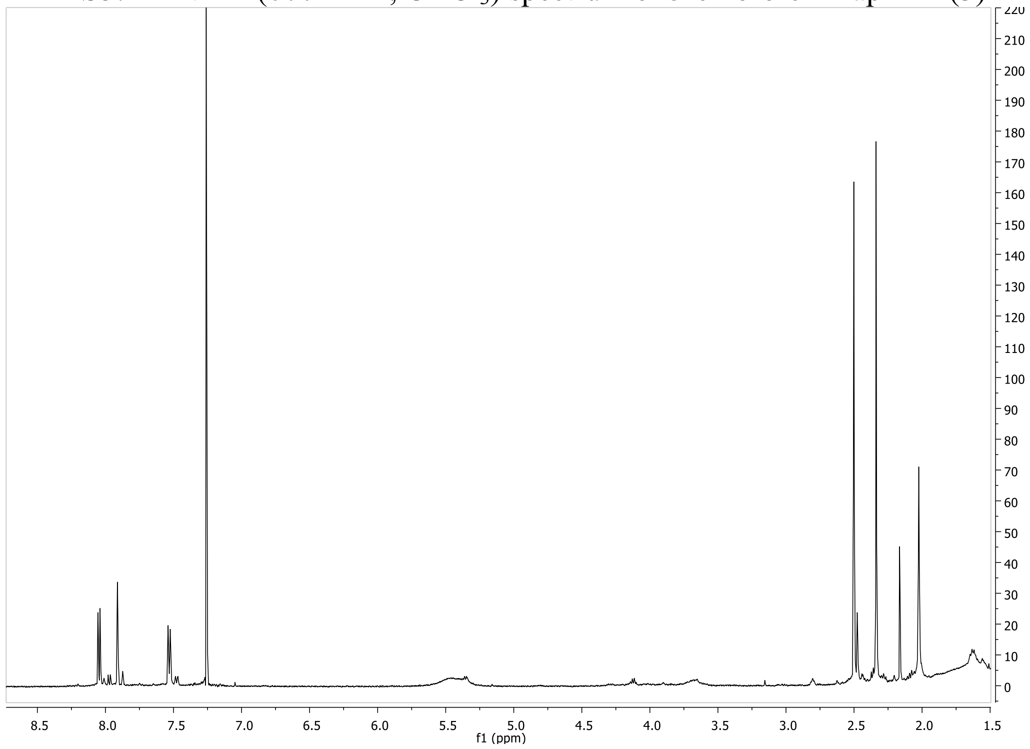


**S6:** $^{13}$C NMR (125 MHz, $CDCl_3$) spectrum for 3-chloro-chimaphilin (**3**)

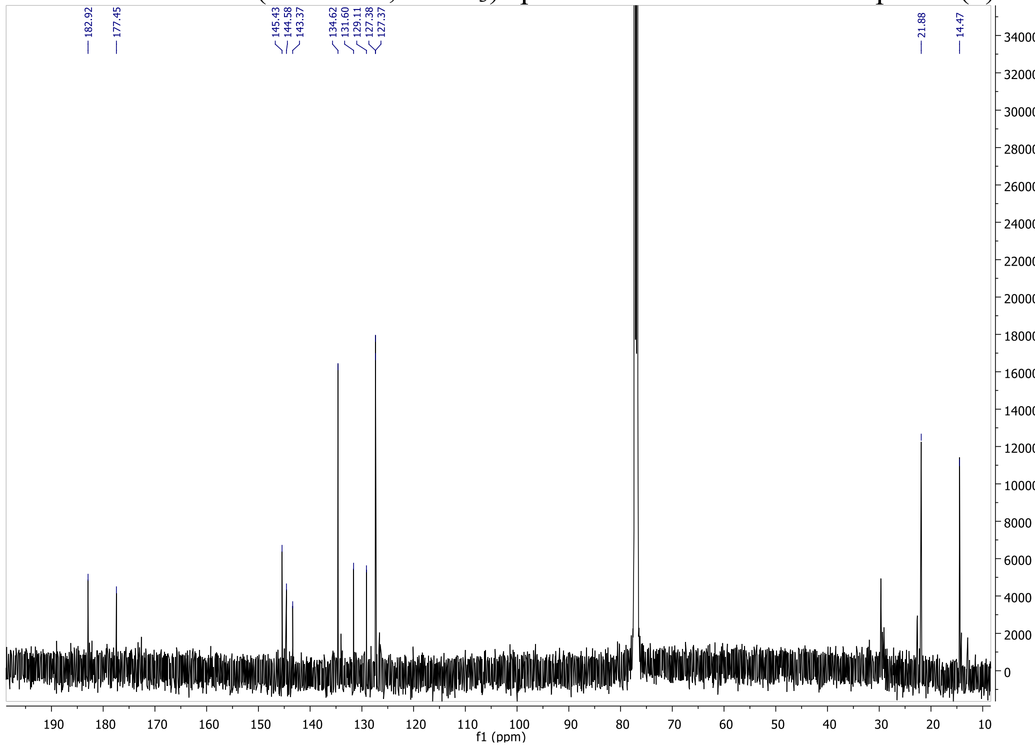

**S7:** gHSQC ($^1$H 500 MHz, $CDCl_3$) spectrum for 3-chloro-chimaphilin (**3**)

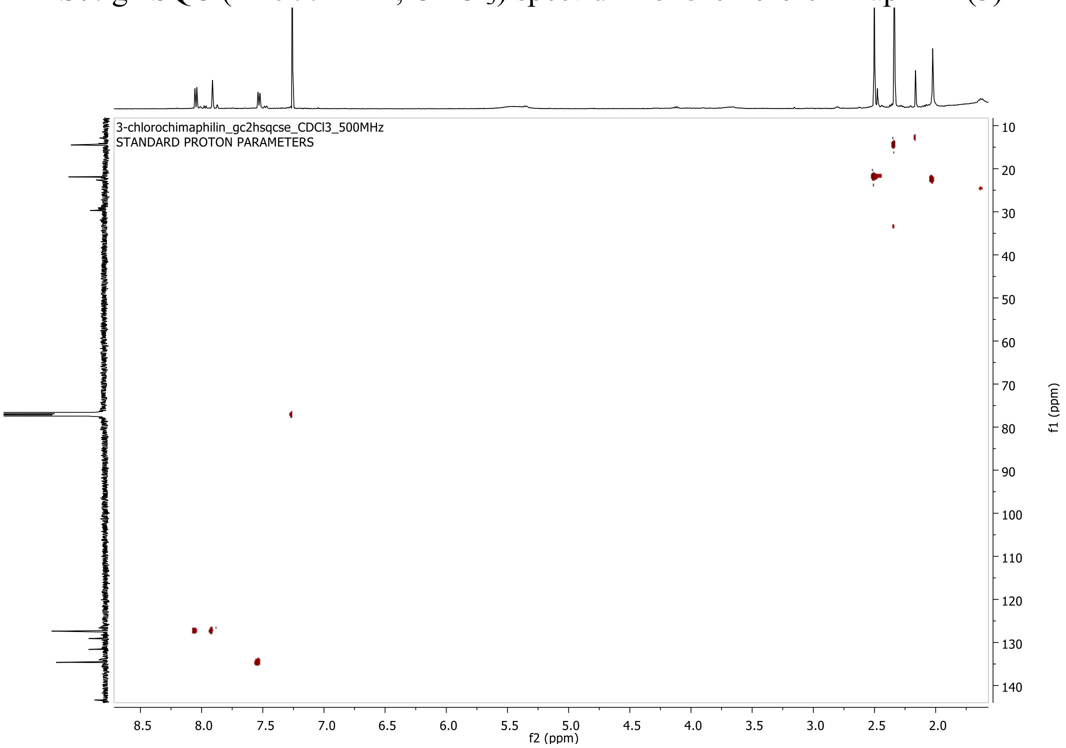


**S8:** gCOSY ($^1$H 600 MHz, $CDCl_3$) spectrum for 3-chloro-chimaphilin (**3**)

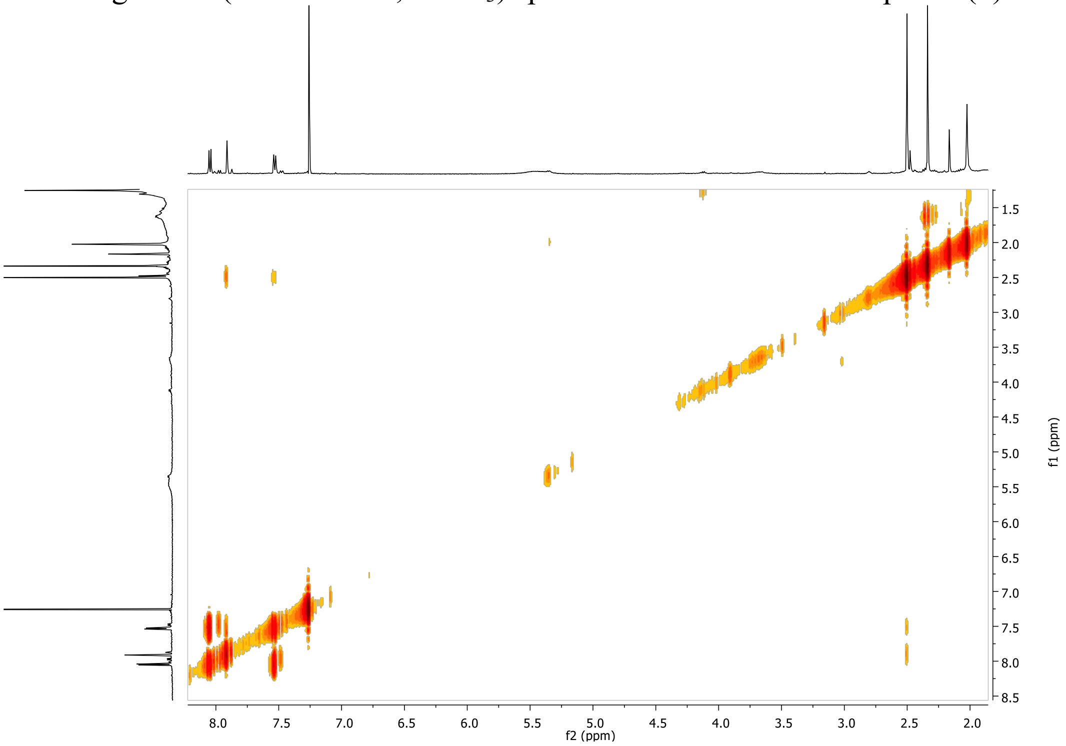

**S9:** gHMBCAD ($^1$H 500 MHz, $CDCl_3$) spectrum for 3-chloro-chimaphilin (**3**)

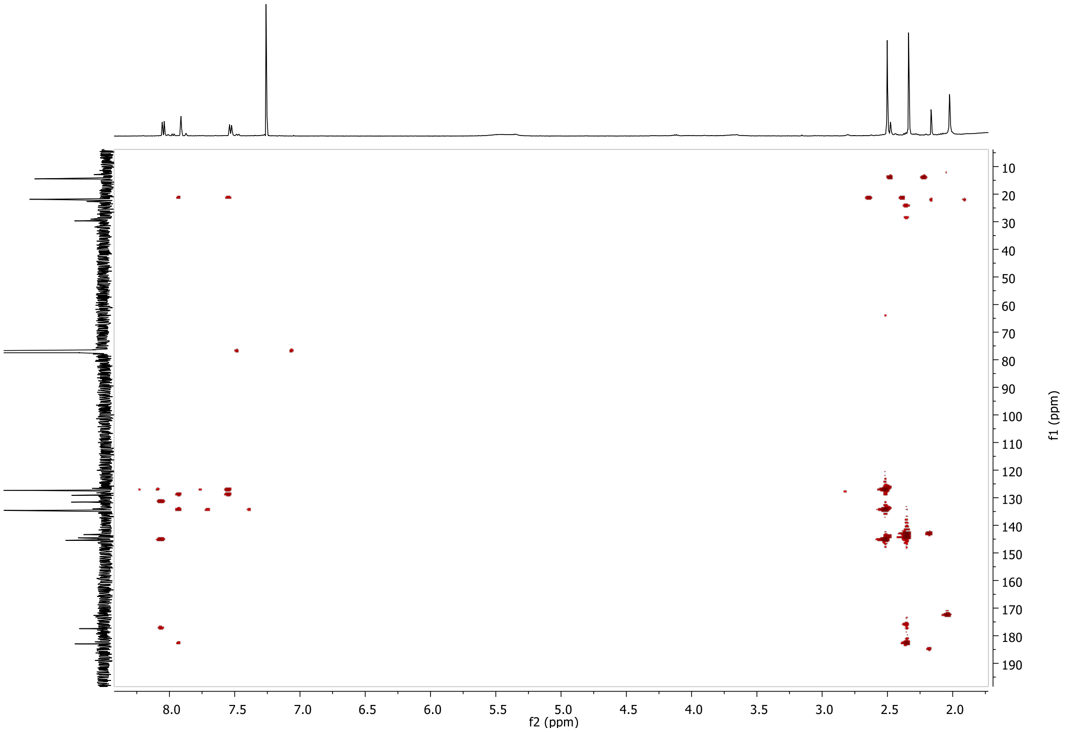


**S1:** $^1$H and $^{13}$C NMR data table for 3-chloro-chimaphilin (**3**) in $CDCl_3$

| **position** | **$\delta_C$ [b]** | **$\delta_H$ (*J* in Hz)[a]** | **HMBC[a]** | **COSY[c]** |
|---|---|---|---|---|
| 1 | 182.9 | | | |
| 2 | 143.4[d] | | | |
| 3 | 144.6[d] | | | |
| 4 | 177.4 | | | |
| 5 | 131.6 | | | |
| 6 | 127.4 | 8.05 (d; 7.8) | 4, 5, 10 | 7 |
| 7 | 134.6 | 7.53 (d; 7.8) | 6, 8, 8-Me | 6, 8-Me, 9 |
| 8 | 129.1 | | | |
| 9 | 127.4 | 7.91 (s) | 1, 7, 8, 8-Me | 7, 8-Me |
| 10 | 145.4 | | | |
| 2-Me | 14.5 | 2.34 (s) | 1, 2, 3, 4 | |
| 8-Me | 21.9 | 2.50 (s) | 7, 9, 10 | 7, 9 |

[a]500 MHz for $^1$H NMR, gHMBCAD, [b]125 MHz for $^{13}$C NMR, [c]600 MHz for gCOSY
[d]Ambiguous assignment

**S2:** $^{1}$H and $^{13}$C NMR assignments for chimaphilin (**1**), 3-chloro-chimaphilin (**2**), and 3-hydroxy-chimaphilin (**3**) in $CDCl_3$

| Cmpd | Chimaphilin | | 3-chlorochimaphilin | | 3-hydroxychimaphilin | |
|---|---|---|---|---|---|---|
| Position | $\delta_C$ [c] | $\delta_H$ [a] | $\delta_C$ [d] | $\delta_H$ [b] | $\delta_C$ [c] | $\delta_H$ [a] |
| 1 | 184.5 | | 182.9 | | 185.5 | |
| 2 | 148.2 | | 143.4[d] | | 145.2 | |
| 3 | 144.6 | 6.69 (s) | 144.6[d] | | 153.5 | |
| 4 | 185.9 | | 177.4 | | 181.5 | |
| 5 | 126.2 | | 131.6 | | 126.3 | |
| 6 | 135.7 | 7.82 (d; 7.8) | 127.4 | 8.05 (d; 7.8) | 132.8 | 7.97 (d; 7.7) |
| 7 | 134.3 | 7.42 (d; 7.8) | 134.6 | 7.53 (d; 7.8) | 133.4 | 7.46 (d; 7.7) |
| 8 | 126.8 | | 129.1 | | 127.2 | |
| 9 | 132.0 | 7.77 (s) | 127.4 | 7.91 (s) | 127.0 | 7.91 (s) |
| 10 | 131.1 | | 145.4 | | 120.0 | |
| 2-Me | 16.4 | 2.06 (s) | 14.5 | 2.34 (s) | 8.64 | 2.09 (s) |
| 8-Me | 21.8 | 2.38 (s) | 21.9 | 2.50 (s) | 22.06 | 2.49 (s) |

[a]600 MHz, [b]150 MHz, [c]125 MHz
[d]Ambiguous assignment

**S10:** HRESIMS spectrum for chimaphilin (**1**)

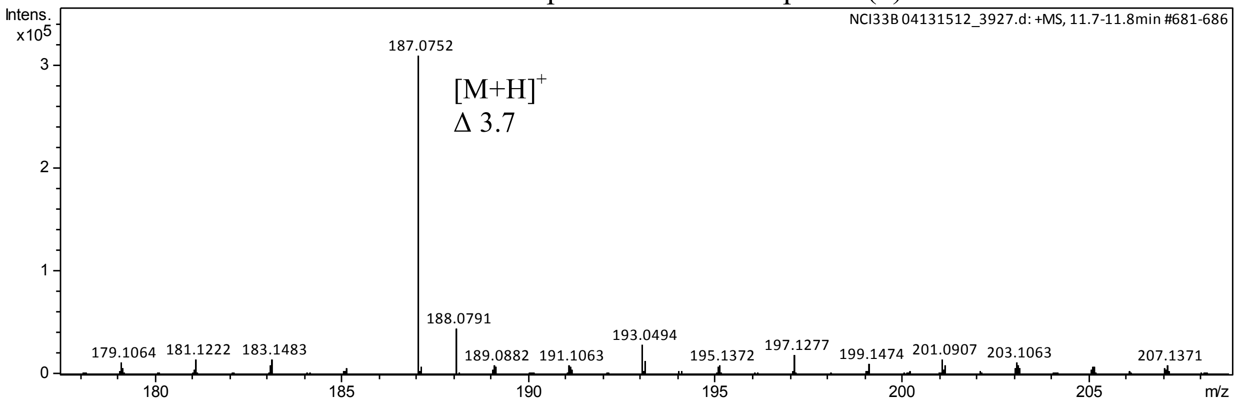


**S11:** HRESIMS spectrum for 3-hydroxy-chimaphilin (**2**)

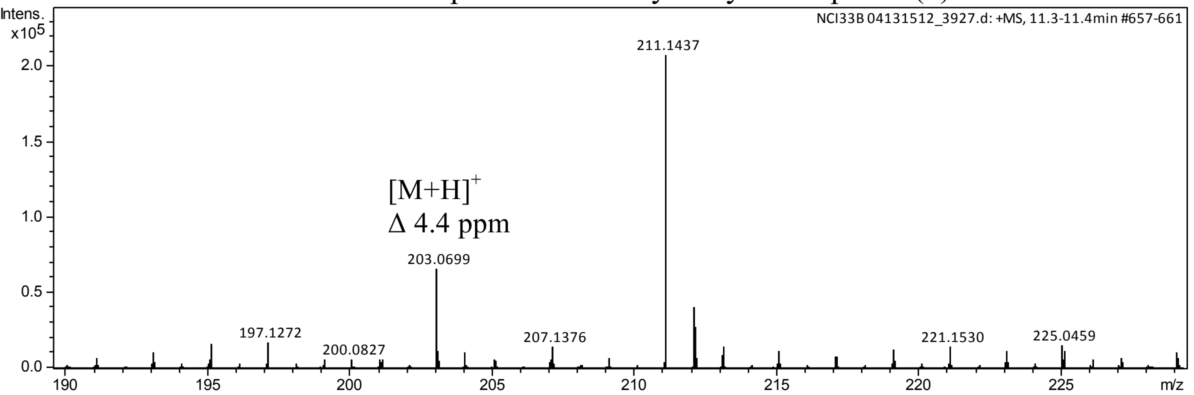

**S12:** HRESIMS spectrum for 3-chloro-chimaphilin (**3**)

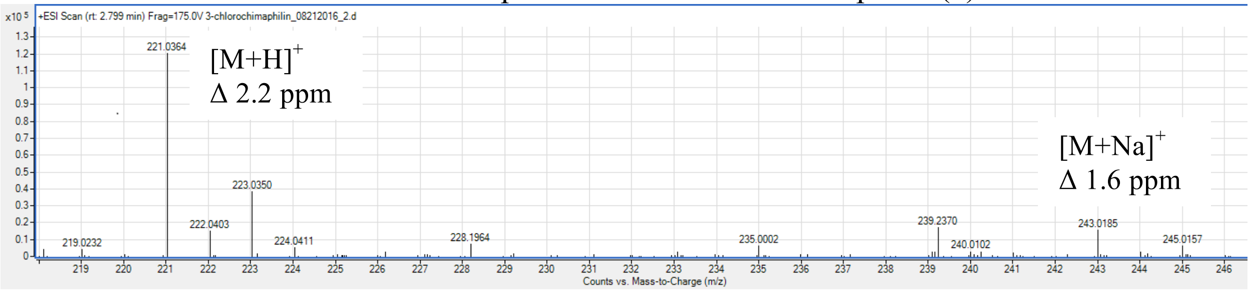

Supplement: S1 Appendix — (PDF) [file pone.0162686.s001.pdf]
