# Supplementary material for: Natural Product Screening Reveals Naphthoquinone Complex I Bypass Factors
Source: PLoS One. 2016 Sep 13;11(9):e0162686. doi: 10.1371/journal.pone.0162686 (PMC5021346; doi:10.1371/journal.pone.0162686)

S2 Appendix

Analytical Data for the Lapachone Family of Metabolites

NMR Spectroscopic Data:

Figure S1 ^{1}H NMR (600 MHz, $CDCl_3$) spectrum of lapachol (**4**)
Figure S2 ^{13}C NMR (150 MHz, $CDCl_3$) spectrum of lapachol (**4**)
Figure S3 ^{1}H NMR (600 MHz, $CDCl_3$) spectrum of dehydro-α-lapachone (**5**)
Figure S4 ^{13}C NMR (150 MHz, $CDCl_3$) spectrum of dehydro-α-lapachone (**5**)
Figure S5 ^{1}H NMR (600 MHz, $CDCl_3$) spectrum of α-lapachone (**6**)
Figure S6 ^{13}C NMR (125 MHz, $CDCl_3$) spectrum of α-lapachone (**6**)
Figure S7 ^{1}H NMR (600 MHz, $CDCl_3$) spectrum of dehydroiso-α-lapachone (**7**)
Figure S8 ^{13}C NMR (125 MHz, $CDCl_3$) spectrum of dehydroiso-α-lapachone (**7**)
Figure S9 gHSQC (^{1}H 500 MHz, $CDCl_3$) spectrum for dehydroiso-α-lapachone (**7**)
Figure S10 gCOSY (^{1}H 500 MHz, $CDCl_3$) spectrum for dehydroiso-α-lapachone (**7**)
Figure S11 gHMBC (^{1}H 500 MHz, $CDCl_3$) spectrum for dehydroiso-α-lapachone (**7**)
Table S1 ^{1}H and ^{13}C NMR data table for dehydroiso-α-lapachone (**7**) in $CDCl_3$

HPLC Chromatograms:

Figure S12 Chiral HPLC of dehydroiso-α-lapachone (**7**)

HRESIMS Data:

Figure S13 HRESIMS spectrum for lapachol (**4**)
Figure S14 HRESIMS spectrum for dehydro-α-lapachone (**5**)
Figure S15 HRESIMS spectrum for α-lapachone (**6**)
Figure S16 HRESIMS spectrum for dehydroiso-α-lapachone (**7**)

S1: ^{1}H (600 MHz, $CDCl_3$) spectrum for lapachol (**4**)

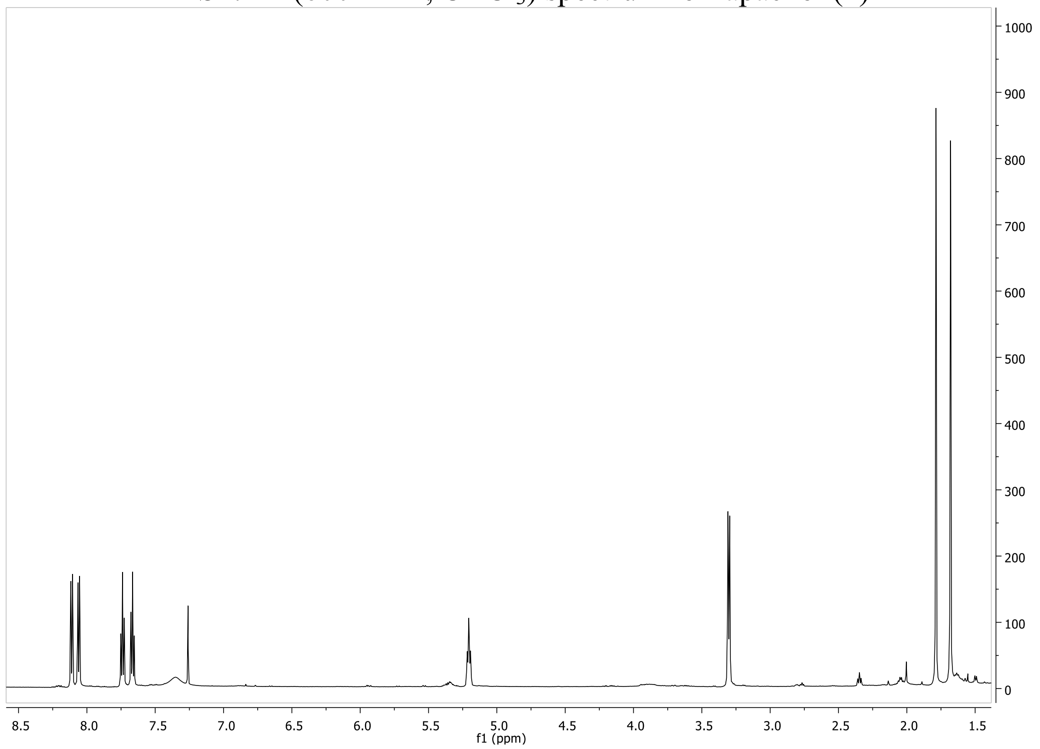

S2: ^{13}C (150 MHz, $CDCl_3$) spectrum for lapachol (**4**)

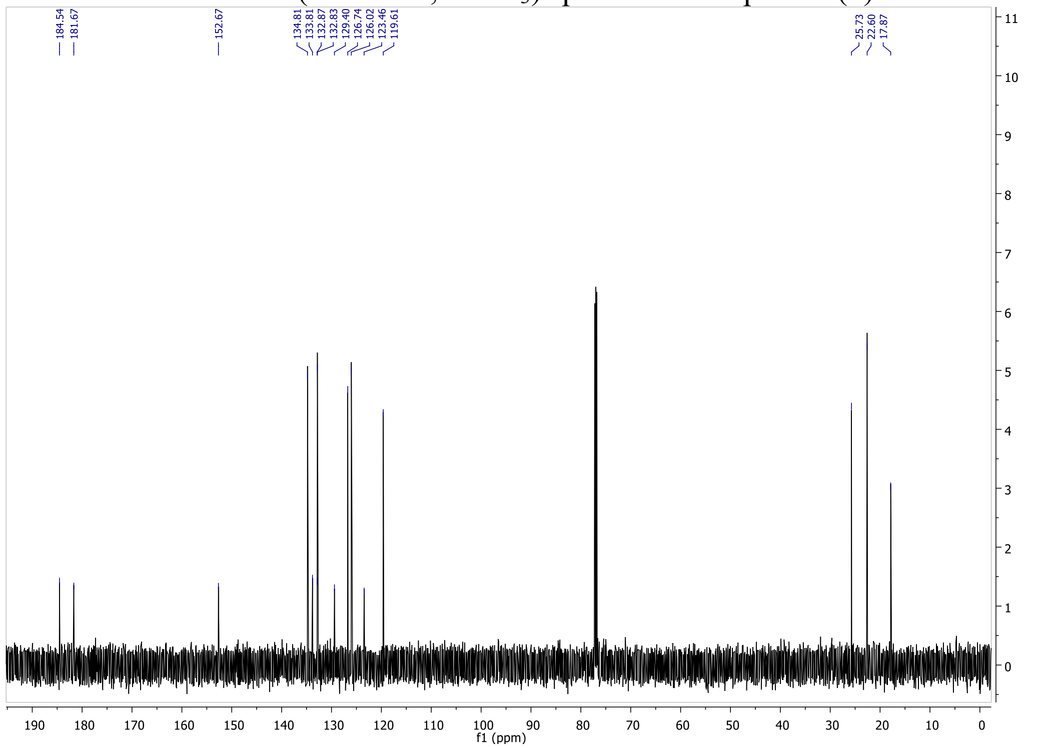

S3: ^{1}H (600 MHz, $CDCl_3$) spectrum for dehydro-α-lapachone (**5**)

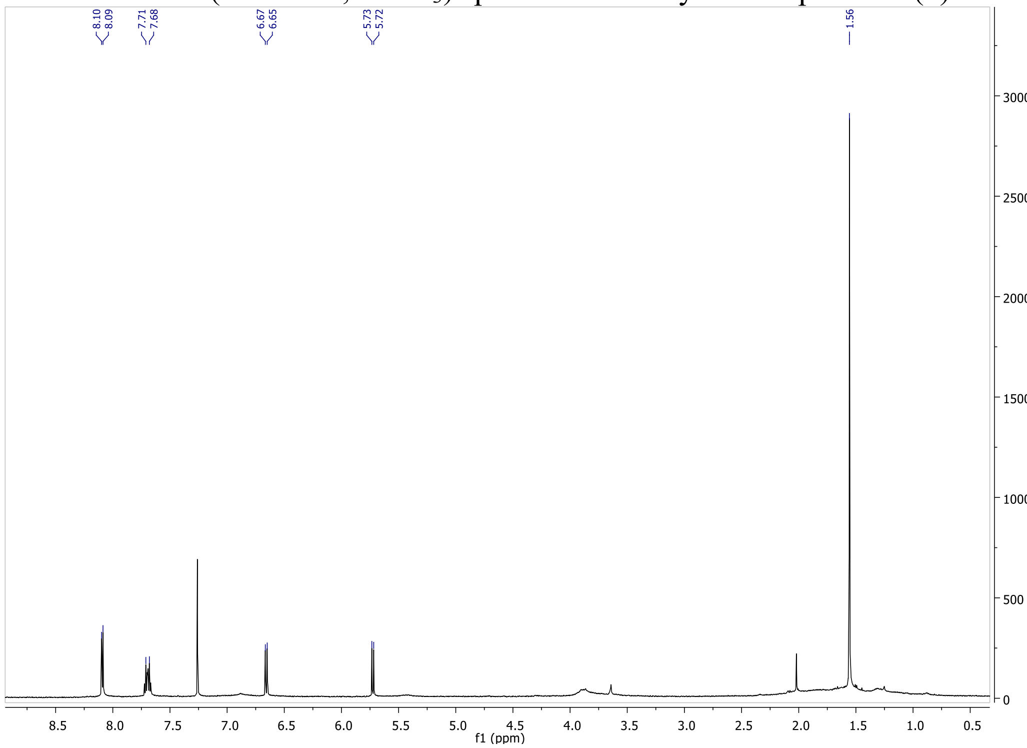

S4: ^{13}C (150 MHz, $CDCl_3$) spectrum for dehydro-α-lapachone (**5**)

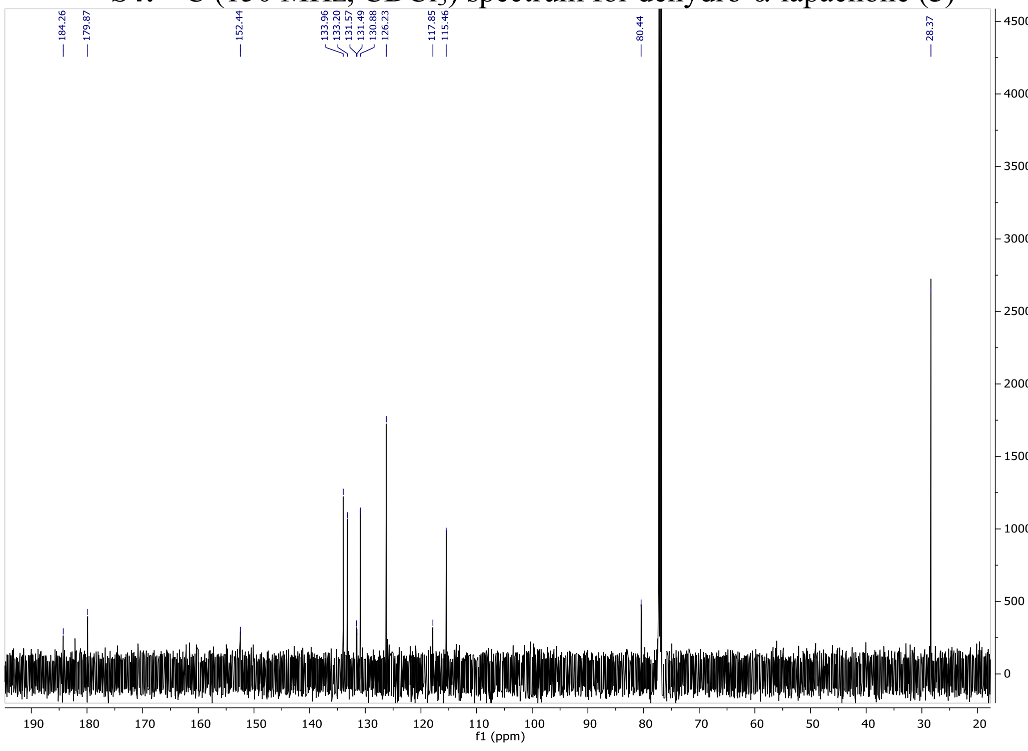

S5: ^{1}H (600 MHz, $CDCl_3$) spectrum for α-lapachone (**6**)

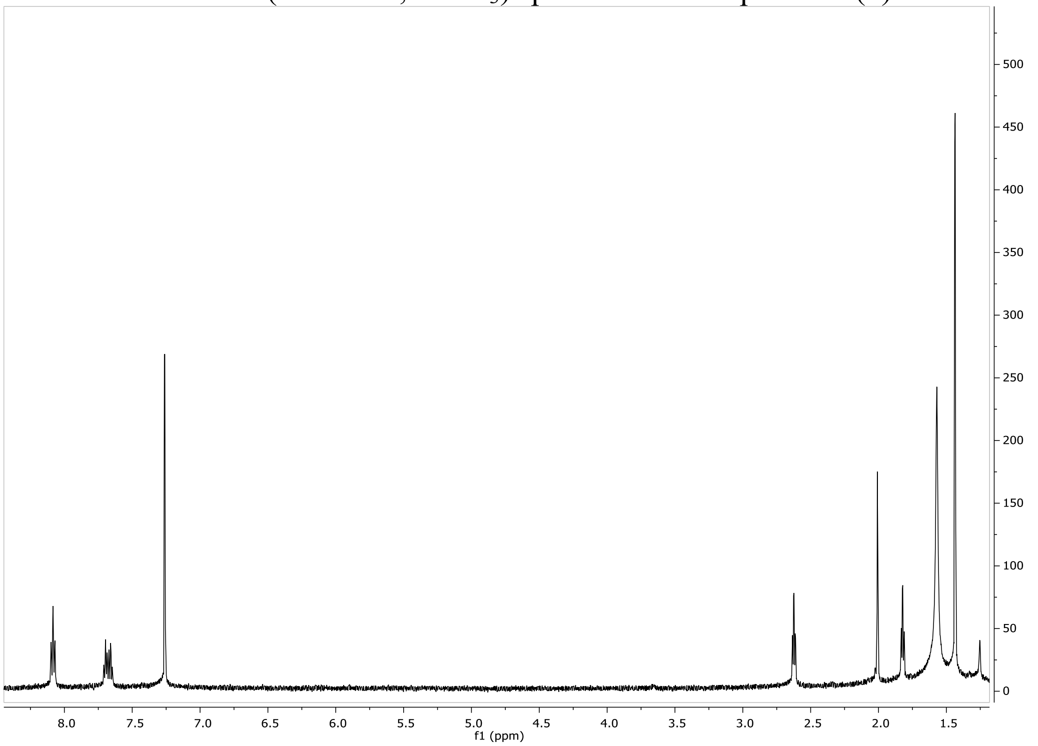

S6: ^{13}C (150 MHz, $CDCl_3$) spectrum for α-lapachone (**6**)

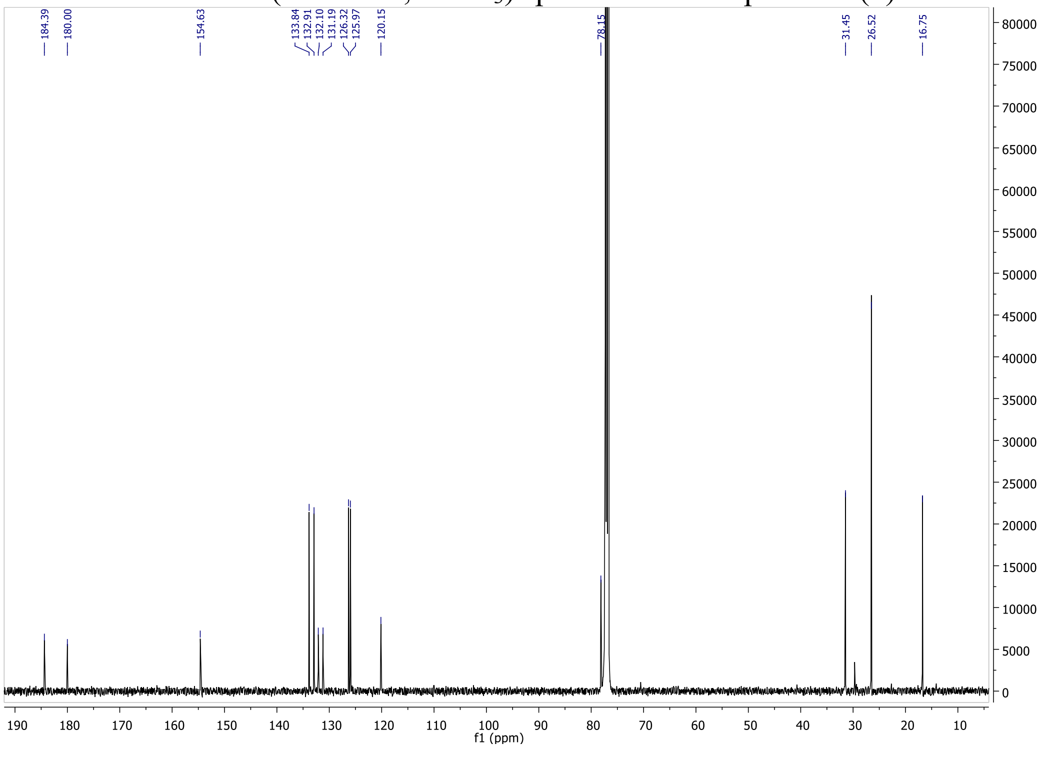

S7: ^{1}H (600 MHz, $CDCl_3$) spectrum for dehydroiso-α-lapachone (**7**)

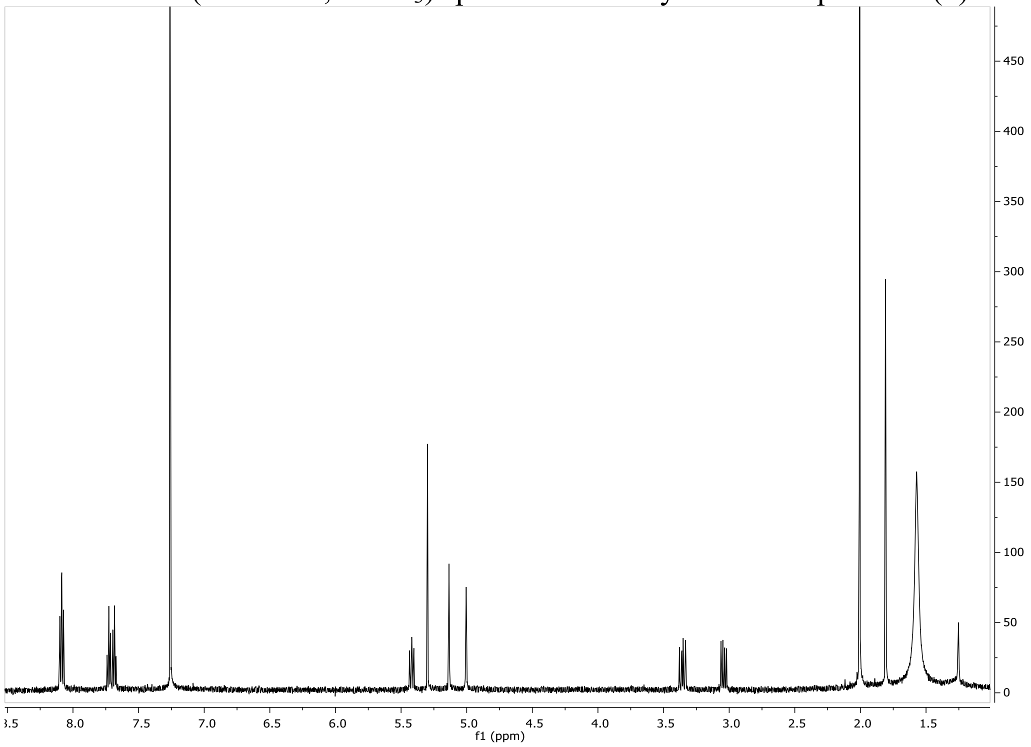

S8: ^{13}C (125 MHz, $CDCl_3$) spectrum for dehydroiso-α-lapachone (**7**)

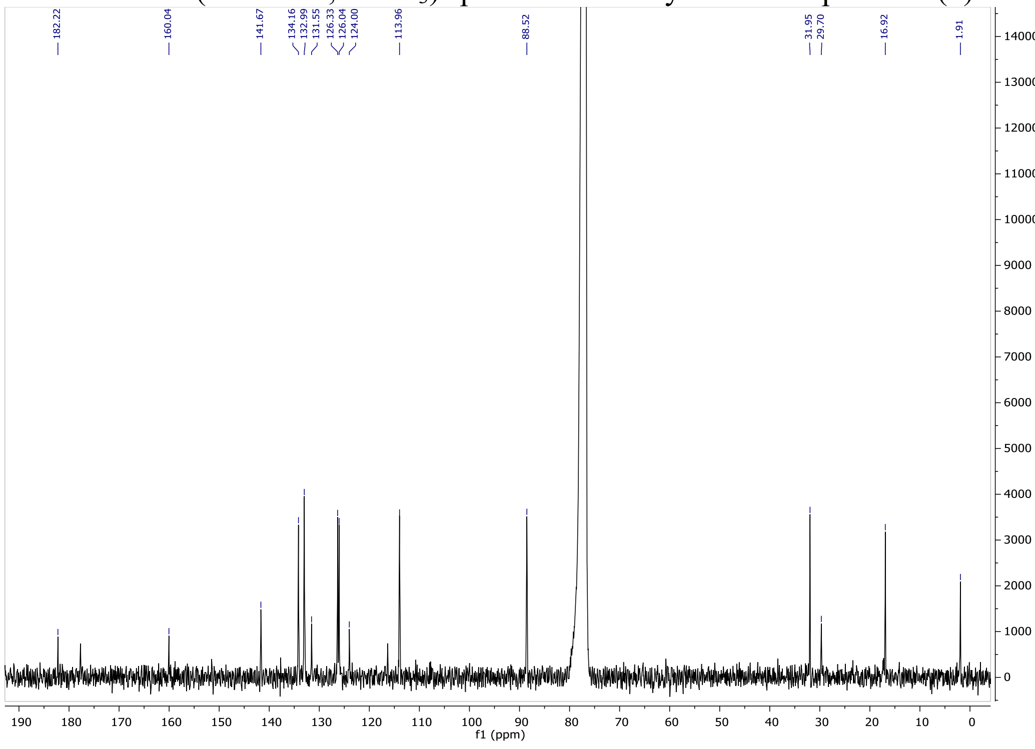

S9: gHSQC (^{1}H 500 MHz, $CDCl_3$) spectrum for dehydroiso-α-lapachone (**7**)

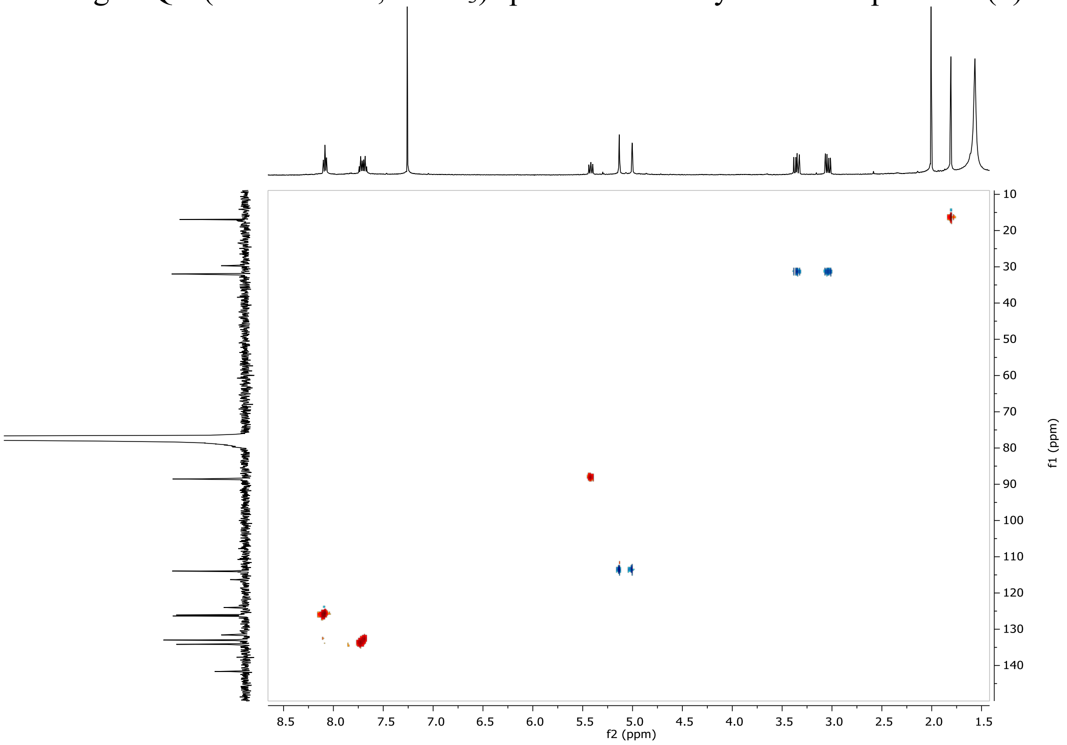

S10: gCOSY (^{1}H 500 MHz, $CDCl_3$) spectrum for dehydroiso-α-lapachone (**7**)

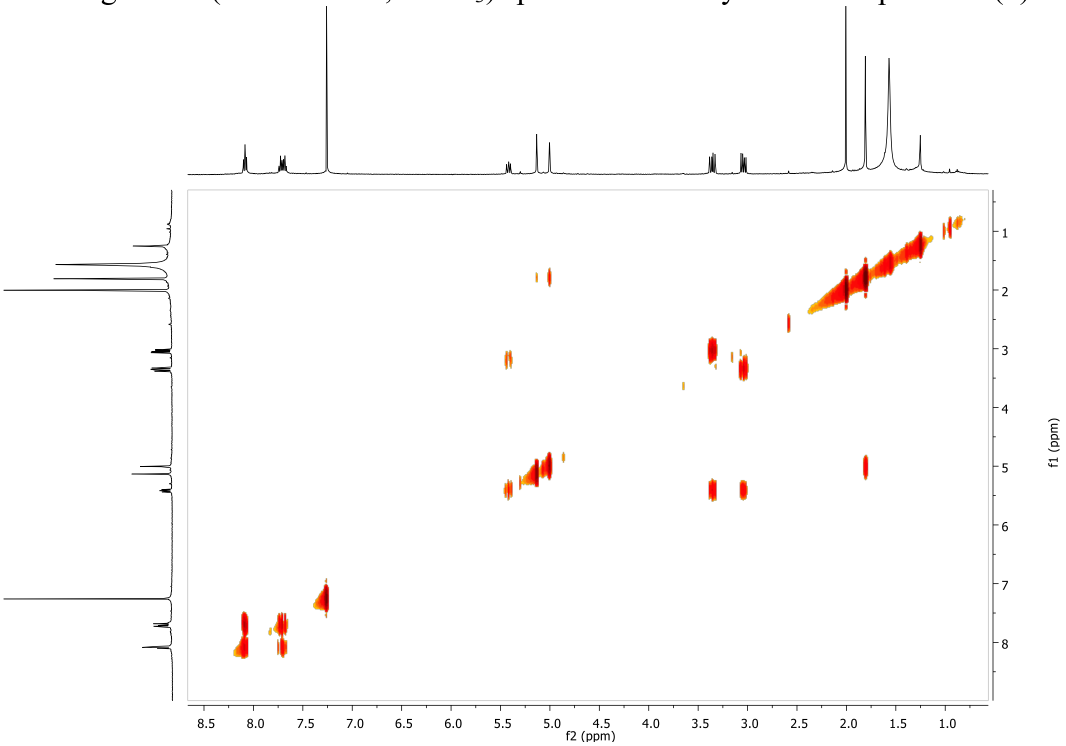

S11: gHMBCAD (^{1}H 500 MHz, $CDCl_3$) spectrum for dehydroiso-α-lapachone (**7**)

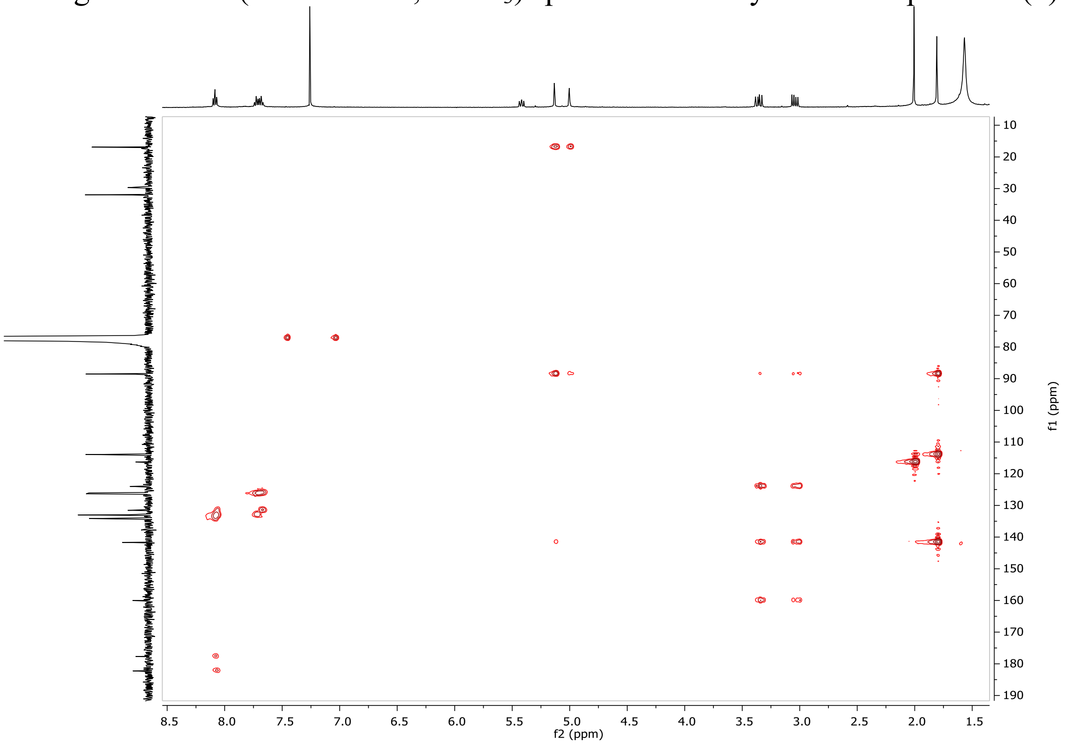

S1: ^{1}H and ^{13}C NMR data table for dehydroiso-α-lapachone (**7**) in $CDCl_3$

**position**	**δ_C [b]**	**δ_H (*J* in Hz) [a]**	**HMBC[a]**	**COSY[a]**
1a	113.9	5.13 (s)	2, 3, 15	15
1b		5.00 (s)	3, 15	15
2	141.6			
3	88.5	5.42 (dd; 10.8, 9.0)	1	4a, 4b
4a	32.0	3.35 (dd; 17.2, 10.8)	2, 5, 6	3, 4b
4b		3.03 (dd; 17.2, 9.0)	2, 3, 5, 6	3, 4a
5	124.0			
6	160.0			
7	177.7			
8	131.6			
9	126.0	8.09 (t; 7.5)	7, 8, 10	10, 11
10	133.0	7.72 (t; 7.5)	9, 11	9/12
11	134.2	7.68 (t; 7.5)	12, 10	9/12
12	126.3	8.09 (t; 7.5)	11, 13, 14	10, 11
13	131.6			
14	182.2			
15	16.9	1.80 (s)	1, 2, 3	1a, 1b

[a]500 MHz for ^{1}H NMR, gHMBCAD, gCOSY, [b]125 MHz for ^{13}C NMR

S12: Chiral HPLC of dehydroiso-α-lapachone (**7**)

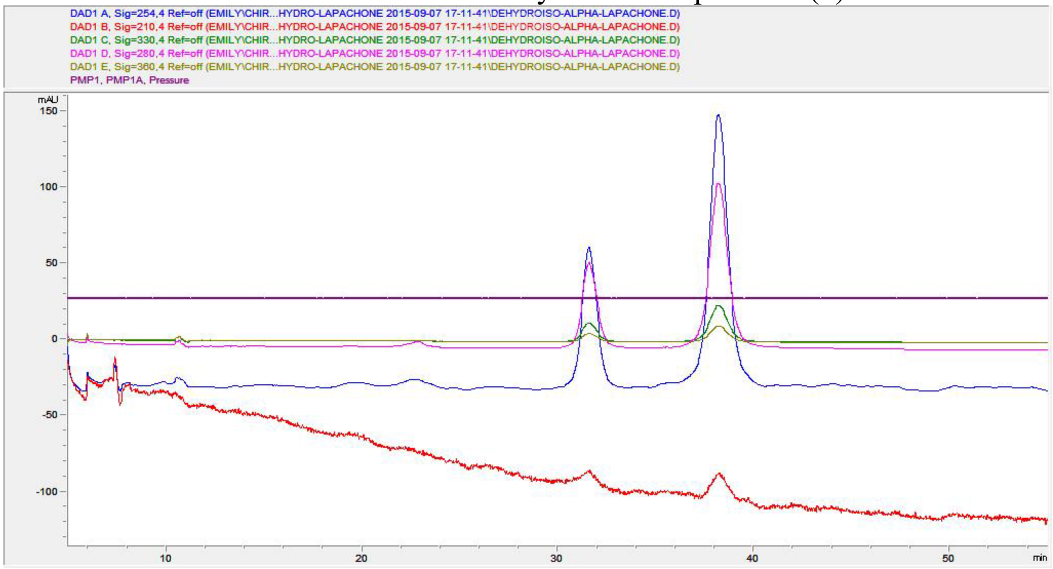

<u>Figure S12:</u> Dehydroiso- α-lapachone (**7**) is a mixture of enantiomers.
<u>HPLC Method:</u> Isocratic gradient using 1% ethanol + 0.1% DEA/hexane + 0.1% DEA with a flow rate of 0.7 mL/min on a Phenomenex Cellulose-4 analytical column (4.6 x 250 mm).

S13: HRESIMS spectrum for lapachol (**4**)

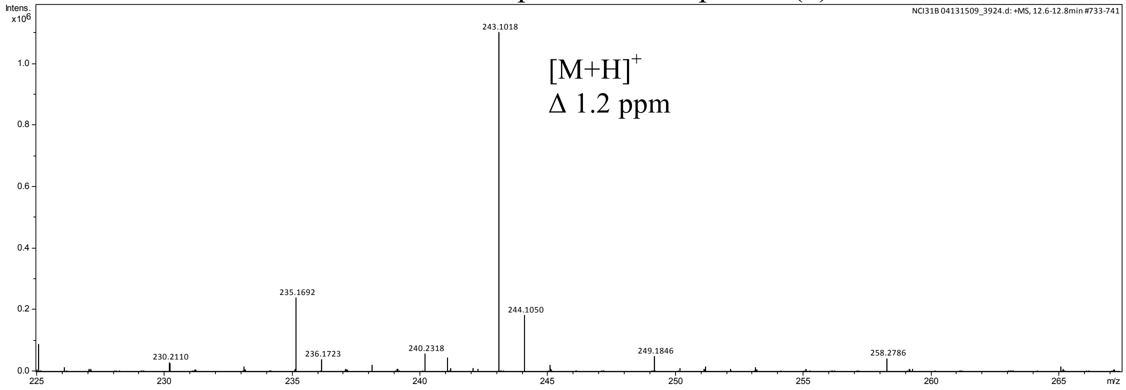

S14: HRESIMS spectrum for dehydro-α-lapachone (**5**)

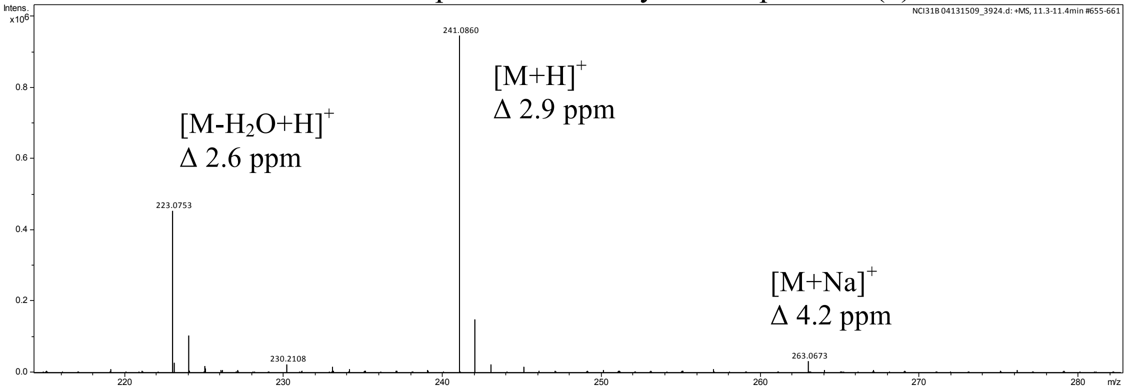

S15: HRESIMS spectrum for α-lapachone (**6**)

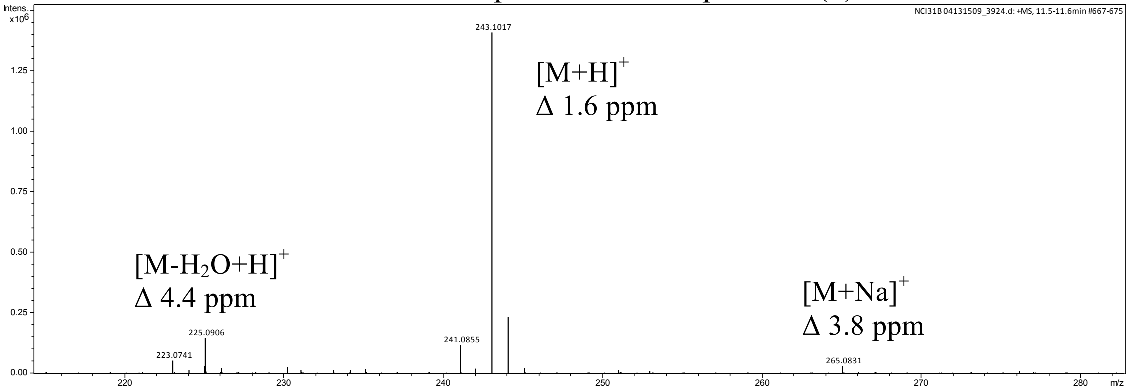

S16: HRESIMS spectrum for dehydroiso-α-lapachone (**7**)

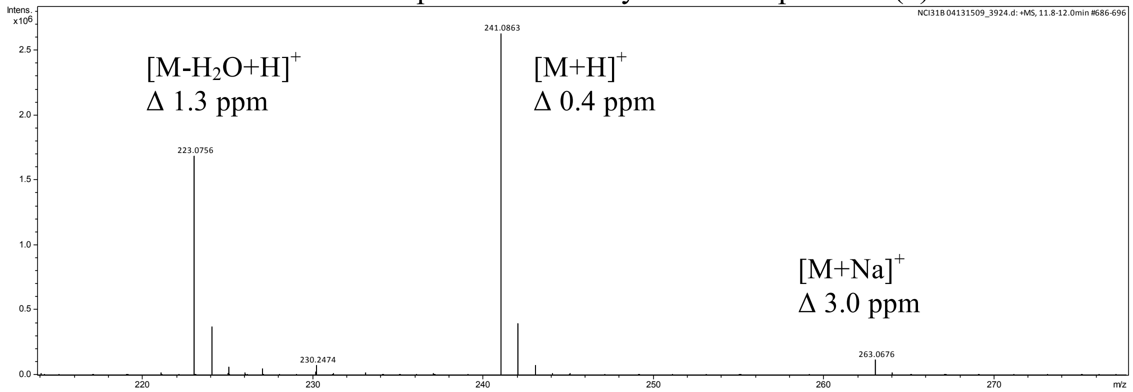

Supplement: S2 Appendix — Also included is the chiral HPLC chromatogram for dehydroiso-α-lapachone (7). (PDF) [file pone.0162686.s002.pdf]
