# Supplementary figures and images for: Natural Product Screening Reveals Naphthoquinone Complex I Bypass Factors
Source: PLoS One. 2016 Sep 13;11(9):e0162686. doi: 10.1371/journal.pone.0162686 (PMC5021346; doi:10.1371/journal.pone.0162686)

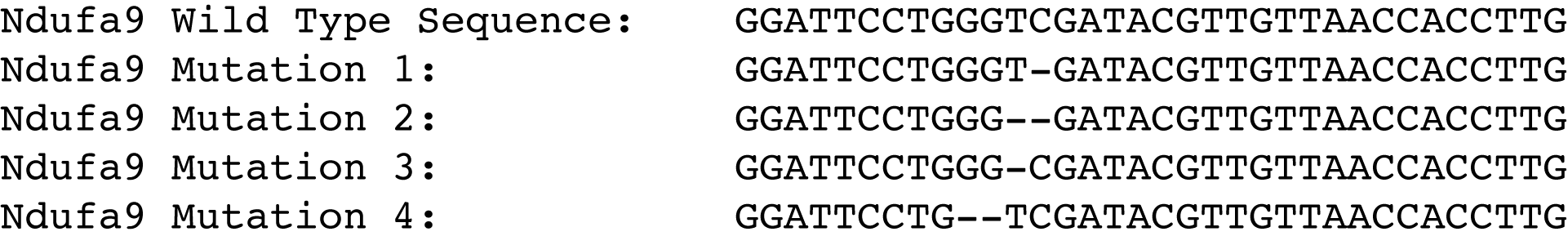

Supplement: S1 Fig — (TIF) [file pone.0162686.s003.tif]

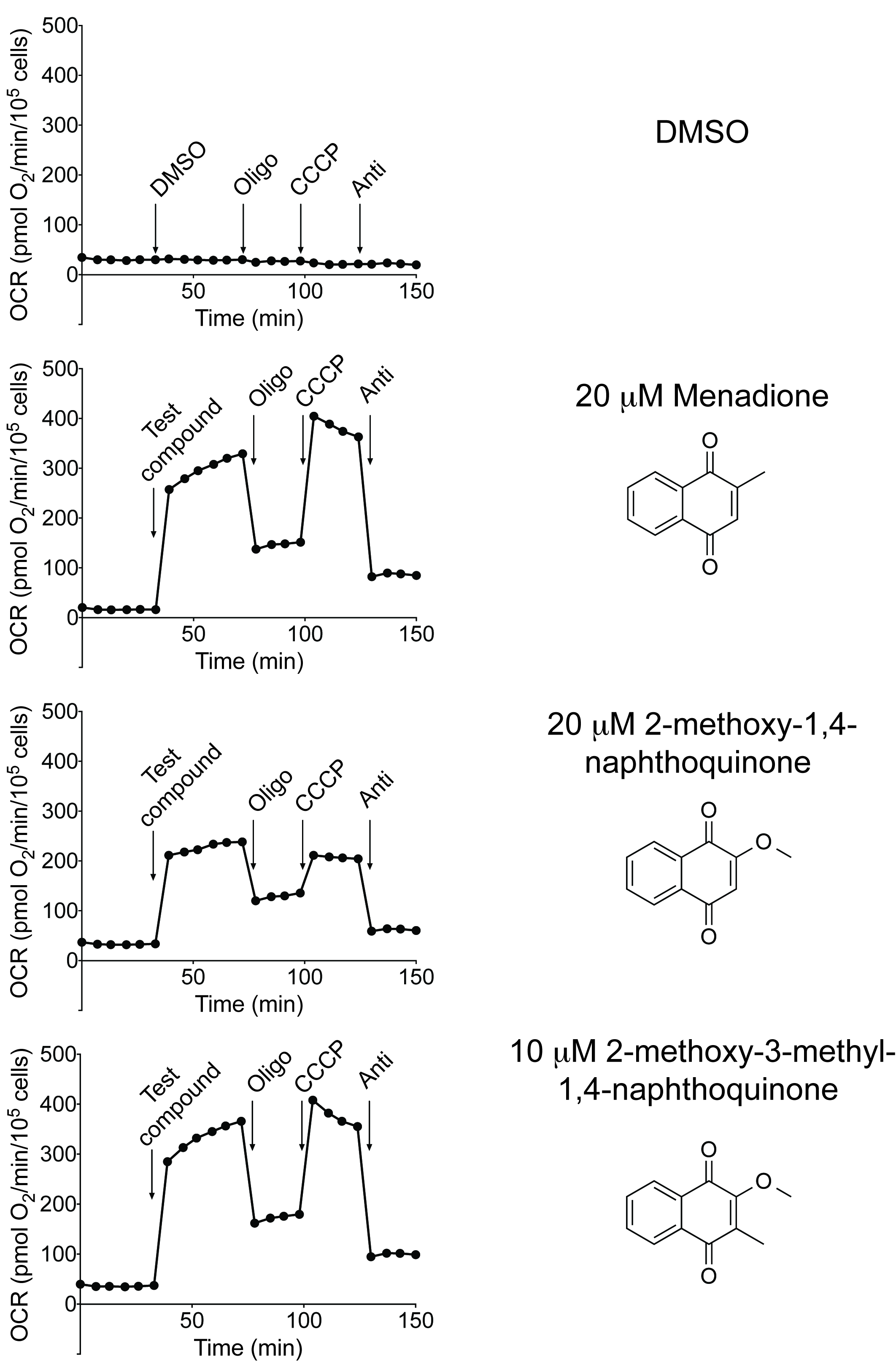

Supplement: S2 Fig — This experiment was performed three times and a single representative experiment is shown. (TIF) [file pone.0162686.s004.tif]
